# Multiple strain analysis of *Streptomyces* species from Philippine marine sediments reveals intraspecies heterogeneity in antibiotic activities

**DOI:** 10.1038/s41598-021-96886-4

**Published:** 2021-09-02

**Authors:** Chuckcris P. Tenebro, Dana Joanne Von L. Trono, Carmela Vannette B. Vicera, Edna M. Sabido, Jovito A. Ysulat, Aaron Joseph M. Macaspac, Kimberly A. Tampus, Trisha Alexis P. Fabrigar, Jonel P. Saludes, Doralyn S. Dalisay

**Affiliations:** 1 Center for Chemical Biology and Biotechnology (C2B2), University of San Agustin, 5000 Iloilo City, Philippines; 2 Center for Natural Drug Discovery and Development (CND3), University of San Agustin, 5000 Iloilo City, Philippines; 3 Department of Chemistry, College of Liberal Arts, Sciences, and Education, University of San Agustin, 5000 Iloilo City, Philippines; 4 Department of Biology, College of Liberal Arts, Sciences, and Education, University of San Agustin, 5000 Iloilo City, Philippines; 5 Balik Scientist Program, Department of Science and Technology, Philippine Council for Health Research and Development (PCHRD), 1631 Bicutan, Taguig City, Philippines

**Keywords:** Biotechnology, Chemical biology, Drug discovery, Ecology, Microbiology, Ocean sciences

## Abstract

The marine ecosystem has become the hotspot for finding antibiotic-producing actinomycetes across the globe. Although marine-derived actinomycetes display strain-level genomic and chemodiversity, it is unclear whether functional traits, *i.e*., antibiotic activity, vary in near-identical *Streptomyces* species. Here, we report culture-dependent isolation, antibiotic activity, phylogeny, biodiversity, abundance, and distribution of *Streptomyces* isolated from marine sediments across the west-central Philippines. Out of 2212 marine sediment-derived actinomycete strains isolated from 11 geographical sites, 92 strains exhibited antibacterial activities against multidrug-resistant *Staphylococcus aureus*, *Pseudomonas aeruginosa*, and *Escherichia coli*. The 16S rRNA and *rpoB* gene sequence analyses confirmed that antibiotic-producing strains belong to the genus *Streptomyces*, highlighting *Streptomyces parvulus* as the most dominant species and three possible new species. Antibiotic-producing *Streptomyces* strains were highly diverse in Southern Antique, and species diversity increase with marine sediment depth. Multiple strains with near-identical 16S rRNA and *rpoB* gene sequences displayed varying strength of antibiotic activities. The genotyping of PKS and NRPS genes revealed that closely related antibiotic-producing strains have similar BGC domains supported by their close phylogenetic proximity. These findings collectively suggest *Streptomyces*' intraspecies adaptive characteristics in distinct ecological niches that resulted in outcompeting other bacteria through differential antibiotic production.

## Introduction

The substantial increase of antimicrobial abuse in medical and livestock settings has led to the rapid evolution of resistance in pathogens resulting in persistence and high transmission of severe infectious disease^1^. Antimicrobial resistance (AMR) has become a global public health concern. It poses an extreme progressive risk causing 50,000 deaths annually in Europe and the United States alone, and it is estimated to increase to 10 million deaths by 2050^2^. The AMR crisis further worsened as the shortage of antibiotics under the drug development pipeline has become apparent. In 2017, the World Health Organization (WHO) reported 51 antibiotics (including combinations of commercially known drugs) and 11 biologicals in the clinical pipeline. However, given the low success rate, it is projected that approximately only ten potential antibiotics can reach the market in the next ten years^3^. Thus, the AMR crisis indicates the need to identify new antibiotics to fight against multidrug-resistant pathogens.

*Streptomyces* produce a wealth of structurally diverse antibiotics, which are essential in medicine and agriculture due to the emergence of multidrug-resistant pathogens^4–7^. These antibiotics are produced by the biosynthetic gene clusters (BGCs) that are harbored in *Streptomyces* genomes^7,8^. The majority of the identified BGCs sequenced in *Streptomyces* are non-ribosomal peptide synthetase (NRPS), polyketide synthase (PKS), terpenes, lantipeptides, and other ketide synthases^9^^.^ However, only a small fraction of these compounds is produced by strains cultivated in the laboratory, suggesting that each strain's vast chemical entities are still poorly characterized particularly those in underexplored ecological niches. Recent genomic and metabolomics studies provided substantial evidence on genomic and chemodiversity within and between *Streptomyces* species. For example, bioinformatics analysis of six antibiotic-producing *S. albus* strains uncovered 16 strain-specific gene clusters encoding strain-specific secondary metabolites. These findings demonstrate that each strain of *S. albus* likely harbors at least one-specific biosynthetic gene cluster^10^. Moreover, studies on multiple species of antibiotic-producing *S. cyaneofuscatus*^11^ and *S. rimosus*^12^ also disclosed strain-level BGC genomic variation and metabolomics diversity among closely related strains. Recently, genome mining on the most extensive BGC study to date using 1110 publicly available *Streptomyces* genomes revealed a high diversity of BGCs and distribution pattern variation in closely related strains of *Streptomyces*^9^. This BGCs variation in *Streptomyce*s species is associated with an intra- and interspecies recombination as a rapid response to selective pressures in ecological niches, thus impacting the secondary metabolite diversity among closely related strains^8, 13–16^. However, it remains unclear if functional trait (*i.e*., antibiotic activities) varies in a population of closely related strains of *Streptomyces* species as previous studies were all based on genome-wide analysis and metabolite profiling of *Streptomyces* species available in the database. There is no consensus on the distribution, co-occurrence, and antibiotic activity variations among multiple *Streptomyces* species and taxonomically closely related strains isolated from the same ecological niche, *i.e.*, marine sediments. To date, strain comparison studies are limited to identical *Streptomyces* strains originating from distant geographic regions. Four strains of *Streptomyces pratensis* isolated from edaphical similar grass fields in New York and North Carolina revealed high level conservation in their core genome, and genomic variation due to recombination^13^. Meanwhile, metabolomics study of *S. griseus* strains isolated from Easter Island and Russia's remote locations showed strain-specific pigmentation and accessory secondary metabolite production. However, both strains exhibited almost similar antibiotic activities against Gram-positive and Gram-negative pathogens^17^.

The marine sediment ecosystem has been recognized as a hotspot for finding antibiotic-producing bacteria across the globe in the last five years^18–21^. *Streptomyces* constitute a minor yet widespread component in marine sediment communities. They are either obligate marine or terrestrial strains that were washed to the shore. The unique physical properties of marine ecosystems such as high salinity and pressure, variation in temperatures, and oxygen concentrations serve as evolutionary pressures that trigger secondary metabolites' production different from their terrestrial counterparts^22^.

The marine ecosystem in Philippine archipelago is a biogeographic realm with varied oceanogeographic features (*i.e.*, upwelling systems, anoxic basins, eutrophic coastal areas, and tectonically active regions) that provide a different niche for diverse marine microbial communities^23^. It was reported that the Philippine waters hold a diverse community of fishes, invertebrates, plants, and zooplankton, with which microorganisms may coevolve^23–25^. During the course of our biological and chemical screening of marine sediment-derived actinomycetes from the Philippines as a potential source of antibiotic leads, we recently reported new *Streptomyces* species producing anthracycline and angucycline antibiotics against multidrug-resistant *Staphylococcus aureus*^26, 27^. These recent findings of bioactive compounds produced by *Streptomyces* strains from Philippine marine sediments signify the importance of investigating the diversity and bioactivities of *Streptomyces* species at a larger scope. In the present study, we assembled a culture collection of 2212 marine sediment-derived actinomycete strains isolated by culture-dependent method across the west-central Philippine archipelago. We identified 92 antibiotic-producing *Streptomyces* strains against four pathogens, which were further investigated for phylogenetic analysis using 16S rRNA and *rpoB* (RNA polymerase beta subunit) genes and PCR detection of BGCs. Here, we report the first large-scale study on multiple strain analysis that demonstrated strain-specific antibacterial and anticancer capabilities of phylogenetically identical *Streptomyces* strains isolated from the same ecological niche, *i.e.,* marine sediments. This work provides a foundation for the new paradigm of mining closely related *Streptomyces* species strains from underexplored ecological niches for antibiotic drug discovery.

## Materials and methods

### Environmental sampling

Actinomycetes inhabiting the marine sediments from 11 geographically remote sites in west-central Philippines were investigated, considering that the collection sites must be 1) away from the anthropogenic activities and populated area, 2) no nearby river, and 3) accessible to the research team. Marine sediment collection was conducted from 2017 to 2018, specifically between March and June. The sample collection was performed by SCUBA diving at a depth of 15 to 30 m using a 110-cm sediment core sampler. The piston of the core sampler was manually inserted to the seabed for the core barrel to penetrate using a hammering stroke (Supplementary Fig. S1. Informed consent was obtained for the participants in the photo). The 110-cm compact sediment samples were partitioned into five layers at 25-cm increments from the seabed: surface (0–25 cm), subsurface (26–50 cm), middle (51–75 cm), sub-bottom (76–90 cm), and bottom (91–110 cm) sediments. The salinity and pH of seawater were measured using a refractometer and pH meter, respectively. Samples in tubes were secured in a ziplock container, maintained temporarily on ice, and transported immediately to the laboratory for storage at 4 °C until further analysis.

### Culture-dependent actinomycete isolation

Marine sediments were processed in the laboratory using dry stamp (DSM) and heat shock method (HSM) to eliminate undesirable growth of microorganisms other than actinomycete^28^. Sediments were inoculated in different minimal marine media containing the following carbon sources: MM3 (0.5 g mannitol, 0.1 g peptone, 15 g agar and 1 L artificial seawater), MM11 (0.5 g glucose, 0.5 g yeast extract, 1 g peptone, 0.01 g FeSO_4_·7H_2_O, 0.02 g Na_2_HPO_4_, 15 g agar, and 1 L artificial seawater), MM13 (0.5 g trehalose, 0.2 g peptone, 15 g agar and 1 L artificial seawater), MM51 (0.5 g raffinose, 0.2 g peptone, 15 g agar and 1 L artificial seawater) and ISP4 (10 g soluble starch, 1 g K_2_HPO_4_, 1 g MgSO_4_, 1 g NaCl, 2 g (NH_4_)_2_SO_4_, 2 g CaCO_3_, 0.001 g FeSO_4_, 0.001 g MnCl_2_, 0.001 g ZnSO_4_, 15 g agar, and 1 L artificial seawater)^29^. The artificial seawater (26.29 g NaCl, 0.74 g KCl, 6.09 g MgCl_2_·6H_2_O, 3.94 g MgSO_4_·7H_2_O and 0.99 g of CaCl_2_ dissolved in 1 L of distilled water) with a pH of 7.8 was sterilized prior to the preparation of the marine media. After 30 to 60 days of incubation, the growth of actinomycete was recognized by the presence of filamentous hyphae and/or by the formation of tough, leathery colonies that adhered to the agar surface. Actinomycete spores in minimal marine media were transferred to enriched marine medium 1 (MM1) containing 10 g/L starch, 4 g/L yeast extract, 2 g/L peptone, 15 g/L agar and artificial seawater agar plates and were incubated at room temperature (25 °C to 28 °C) to obtain pure culture of isolates.

### Resazurin agar overlay assay for antibacterial screening

Before the molecular identification, actinomycete strains were tested for antibacterial activities against four test pathogens: three Gram-negative bacteria (*E. coli* ATCC 25922, *P. aeruginosa* ATCC 27853 and *E. aerogenes* ATCC 13048) and a multidrug-resistant strain *S. aureus* ATCC BAA-44. Isolates were initially screened using a qualitative approach by resazurin reduction test^30^. Briefly, 14-day old pure actinomycete isolate was inoculated using a sterile toothpick in MM1 agar molded in a well of a 96-well microtiter plate. After seven days of incubation at room temperature (25–28 °C), each isolate was overlaid with 100 µL of 1 × 10^6^ CFU/mL bacterial suspension in 0.8% trypticase soy agar (TSA). Overlaid plates were incubated at 37 °C for 18 h. After incubation, 50 µL blue resazurin dye (0.2 mg/mL in PBS) was added to each well. The plate was incubated again at room temperature for 10 to 20 min, and wells that retained the blue color indicated antibacterial activity, while pink wells indicated no activity against the test pathogens (Supplementary Fig. S2). Wells seeded with 20 μL of tetracycline (10 mg/mL in DMSO) were considered as the positive controls, while wells without any treatment were the negative control. This qualitative assay was performed in three replicates.

### Secondary metabolite extraction and confirmatory antibacterial screening

The actinomycete isolates that showed antibacterial activity were subsequently tested for confirmatory screening. First, each bioactive isolate was grown in 500 mL of MM1 medium, incubated for 14 days at room temperature, and the biomass was extracted with ethyl acetate as described previously^26^. Antibacterial activities of initially screened active actinomycete strains’ crude extracts were quantified using microbroth susceptibility testing as described previously^26^. This assay was performed in three replicates. Strains were confirmed to have antibacterial activities as indicated by at least 50% growth inhibition against the test pathogens.

### Anticancer screening

The human ovarian carcinoma cells A2780 (ECACC 93112519) were cultured and maintained in RPMI-1640 medium supplemented with 10% fetal bovine serum (FBS), incubated in humidified 5% CO_2_ at 37 °C.

The antibiotic-producing *Streptomyces* strains were tested for anticancer activities. The cytotoxic effect of bioactive *Streptomyces* strains was tested using lactate dehydrogenase (LDH) determination assay against human ovarian carcinoma (A2780, ECACC) according to the manufacturer’s instruction of cytotoxicity detection kit (LDH) (Roche, Mannheim, Germany) with modification. Only freshly prepared RPMI-1640 medium with 10% FBS and cells with viability of ≥ 95% was used. Each well of a 96-well plate was added with 50 μL media with the extract (to make 400 μg/mL as final concentration) and 50 μL cell suspension (4 × 10^4^ cells/well). The assay was performed in three replicates. The 96-well plate was incubated at 37 °C with 85–95% relative humidity and 5% CO_2_ for 20 h. After incubation, 50 μL of supernatant from all test wells and controls were transferred into a new 96-well plate. Then, 50 μL of reaction mixture from the LDH kit was added to all test wells and controls. The plates were incubated in the dark covered with foil at room temperature for 20 min. To stop the reaction process, 25 μL of stop solution from the LDH kit was then added. The plate was shaken for 10 s prior to reading its absorbance at 490 nm in a microplate reader (BMG LABTECH, Ortenberg, Germany). Crude extracts with activity of 50% and above against ovarian carcinoma (A2780) were considered as bioactive.

The percent cytotoxicity was calculated as follows:

$$\%Cytotoxicity=\frac{(EV-bS)-(L-bDMSO)}{(H-bH)-(L-bDMSO)}$$

wherein: EV = experimental value, bS = background sample control, L = low control, bDMSO = background DMSO control, H = high control, bH = background high control.

### Genomic DNA extraction

The DNA of the active actinomycete strains was extracted for amplification, sequencing, and analysis of small subunit (16S rRNA) and the beta subunit of RNA polymerase (*rpoB*) gene. Bacterial cells from a 7-day broth culture were briefly washed with phosphate buffer saline (pH = 7.4) solution twice and lysed with 3% Triton X-100 (750 μL) at 37 °C for 4 h for cellular membrane degradation and facilitate release of DNA. Subsequently, 50 μL proteinase K (20 mg/mL) (Vivantis Inc., USA) was added to completely lysed cells by degradation of protein contaminants. DNA was precipitated in ice cold ethanol, and further extracted and cleaned using a DNeasy blood and tissue kit (Qiagen, Germany) according to the manufacturer’s instruction.

### 16S rRNA gene amplification, sequencing and analysis

Extracted genomic DNA of active strains were amplified using 27F (5′-AGAGTTTGATCCTGGCTCAG-3′), 518F (5′-CCAGCAGCCGCGGTAATACG-3′), 800R (5′-TACCAGGGTATCTAATCC-3′) and 1492R (5′-TACGGCTACCTTGTTACGACTT-3′) to target the hypervariable regions V1-3 of the small subunit rRNA (16S rRNA) gene^31^, and RNA polymerase beta subunit (*rpoB*) primers^32^, *rpoB* PF (5′-GAGCGCATGACCACCCAGGACGTCGAGGC-3′) and *rpoB* PR (5′-CCTCGCAGTTGTGACCCTCCCACGGCATGA-3′), for β-subunit of DNA-dependent RNA polymerase gene amplification in a CFX-96 Real-Time System (BIO-RAD, Singapore). The reaction mixture (20 μL contained 10 μL SYBR green, 2 μL forward primer (10 μM for 16S rRNA gene; 20 μM for *rpoB* gene), 2 μL reverse primer (10 μM for 16S rRNA gene; 20 μM for rpoB gene), 2 μL nuclease-free H2O and 80 ng DNA template. Three reaction mixtures were prepared for every DNA sample. The PCR condition was based on PCR protocol previously described^27^. According to the manufacturer's protocol, the amplification products were cleaned using a QIAquick PCR Purification Kit (Qiagen, Germany). PCR samples were sent to 1st BASE DNA (Apical Scientific Sdn. Bhd., Malaysia) for sequencing analysis. Forward and reverse sequences of partial 16S rRNA and *rpoB* genes were manually trimmed using BioEdit Sequence Alignment Editor Software (Freeware, Ca, USA)^33^. The resulting partial 16S rRNA (1200 to 1500 bp) and *rpoB* (750–995 bp) gene sequences were closely related to reference strains under genus *Streptomyces*, and the species-level affiliation of the sequences was validated using sequences from Basic Local Alignment Search Tool (BLAST) server from National Center for Biotechnology Information (NCBI). A phylogenetic tree was created using the 16S rRNA and *rpoB* gene sequences of active strains by maximum likelihood algorithms using 1000 bootstraps replicates in MEGA 7.0 (Pennsylvania State University, PA, USA)^34, 35^. The phylogenetic relationships, antibiotic activities, and detected BGCs of the active strains were visualized and annotated using iTOL 5.6.3 (Biobyte solutions, Heidelberg, Germany)^36^.

### PCR amplification and detection of biosynthetic genes PKS type-I, PKS type-II and NRPS

Primers designed to recognize conserved regions in PKS-I ketosynthase (KS), NRPS adenylation (AD), PKS-II ketosynthase alpha (KSα) and ketosynthase beta (KSβ) were selected to investigate natural product biosynthetic gene diversity of active *Streptomyces* strains (Supplementary Table S1). The 20 μL-reaction mixture for this PCR amplification contained 10 μL SYBR green, 1 μL 10 μM forward and 1 μL 10 μM reverse primers, 1 μL DMSO, and 140 ng DNA template. Three reaction mixes were prepared for every DNA sample. To amplify biosynthetic genes targeting Type I polyketide synthase β-ketoacyl synthase (KS) domains fragments, two sets of primers were used: KSMA-F and KSMB-R; and KS-F and KS-R (Supplementary Table S1)^37^. For the PCR protocol using PKS-I KSMA-F and KSMB-R, the reaction started with an initial denaturation at 95 °C for 5 min, followed by 40 cycles at 95 °C for 1 min, 60 °C for 1 min and 72 °C for 2 min, and a 5-min final extension at 72 °C. For gene amplification using PKS-I KS-F and KS-R primers (Supplementary Table S1), the protocol consisted of an initial denaturation at 95 °C for 15 min, one cycle of 95 °C for 1 min, 65 °C for 1 min and 72 °C for 1 min, followed by 35 cycles of 95 °C for 1 min, 62 °C for 1 min and 72 °C for 1 min, with a 10-min final extension at 72 °C. PKS-II ketoacyl synthase alpha (KSα) and ketoacyl synthase Beta (KSβ) domain fragments were amplified using three sets of primers: KS1-F and KS1-R^38^; KSα and KSβ^39^; and 540F and 1100R (Supplementary Table S1)^40^. PCR protocol in detecting PKS-II domains were as follows: initial denaturation at 95 °C for 5 min; 40 cycles at 95 °C for 1 min, 58 °C for 1 min and 72 °C for 2 min; and a 10-min final extension at 72 °C. Meanwhile, for amplification of AD gene fragments, NRPS A3F and A7R primers^41^ were used (Supplementary Table S1). The PCR condition included an initial denaturation of 95 °C for 5 min, followed by 40 cycles of 95 °C for 30 s and 59 °C for 1.5 min, and 72 °C for 1 min, with a 10-min final extension at 72 °C. The PCR amplicons were visualized in 2% (w/v) agarose gel using a Quantum CX5 Edge Gel Doc automated gel imaging and documentation systems (Vilber Lourmat, Collégien, France).

### Data analysis

Species abundance was measured based on the count of individual strain per species. Species diversity index, richness and evenness was calculated using vegan package in RStudio ver1.2.5042 (https://www.rstudio.com/). The geographical map was created using ggplot2 package in RStudio ver1.2.5042 (https://www.rstudio.com/). The heat maps of the different *Streptomyces* species in this study were generated using ComplexHeatmap package in RStudio ver1.2.5042 (https://www.rstudio.com/). Euclidean distance metric method was used with complete hierarchical clustering for the heat maps.

## Results

### Isolation of marine sediment-derived actinomycetes from west-central Philippines

**Figure 1:**
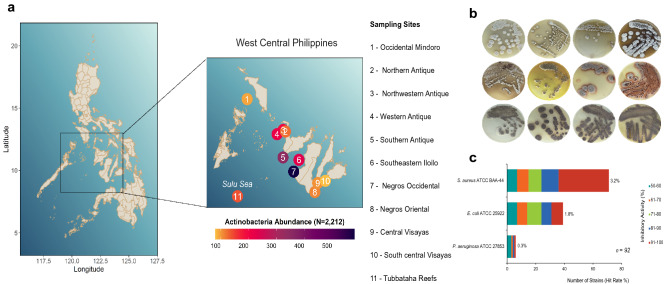
Distribution, abundance, and antibacterial activity of marine sediment-derived actinomycetes in the Philippines. (**a**) The overall map showing the 11 sampling sites situated within the west-central region in the Philippines. The enlarged map showed the details of the sampling sites and their corresponding actinomycetes abundance. Sampling sites are number-coded as shown inside the circle. The color gradient indicates strain abundance. (**b**) Actinomycete colonies were sub-cultured repeatedly to obtain pure culture of isolates. (**c**) A total of 92 out of 2212 actinomycetes strains have confirmed antibacterial activities as verified using microbroth susceptibility assay. The map with *Streptomyces* abundance plot was generated using ggplot2 package in Rstudio ver. 1.2.5042 (https://www.rstudio.com/).

The geographical sites identified in this study were evaluated to explore the actinomycete distribution in west-central Philippines (Fig. 1a). A total of 16 sediment cores were recovered from the 11 sampling sites and were processed in the laboratory using cultured-dependent actinomycete isolation (Supplementary Table S2). The seawater's physicochemical conditions in all sampling sites were uniform with pH 7.0 and salinity ranging from 3.1 to 3.2. The characteristics of marine sediments and the distance of the actual collection sites identified using the given criteria varied per sampling location. The sediment characteristics vary from coarse to fine sand with mixture of broken corals and pebbles (Supplementary Table S3). Culture-dependent isolation revealed that actinomycete-like colonies and spores were observed in the minimal marine media after 30 to 60 days of incubation. Actinomycete isolates were repeatedly sub-cultured in enriched marine medium 1 (MM1) to obtain pure cultures as shown in Fig. 1b. Actinomycete growth observed in enriched media was white to gray aerial spores with brown to yellow mycelia or without diffusible pigmentations. Notably, there were strains that produced colonies with no diffusible pigmentations.

In this work, a total of 2212 pure actinomycete strains were isolated from marine sediments collected in 11 geographically distant sampling sites across the west-central Philippines (Fig. 1a). Actinomycete strains were highly abundant in Negros Occidental with 580 isolates (26%), followed by Southern Antique with 348 isolates (16%) and Southeastern Iloilo with 228 (10%). We recovered least actinomycete strains in Occidental Mindoro and South Central Visayas with only 94 and 81 strains, respectively.

### Antibacterial activity profile of actinomycete strains

We assessed the antibacterial activities of actinomycete strains against a multidrug-resistant Gram-positive bacterium (*S. aureus* ATCC BAA-44) and three Gram-negative bacteria (*E. coli* ATCC 25922, *P. aeruginosa* ATCC 27853, and *E. aerogenes* ATCC 13048) using resazurin agar overlay assay and microbroth susceptibility assay as initial and confirmatory screenings, respectively. A total of 218 (9.9%) out of the 2212 actinomycete isolates have antibacterial activities in the initial screening as indicated by positive results or retained blue resazurin color in wells containing actinomycete overlaid with the test pathogens (Supplementary Fig. S2). The 218 active isolates were fermented to produce biomass for secondary metabolite extraction and to confirm their antibacterial activities by microbroth susceptibility testing. Figure 1c showed the confirmed 92 (4.1%) antibiotic-producing actinomycete strains. The majority of the strains (71 isolates) exhibited activity against Gram-positive *S. aureus* ATCC BAA-44. Thirty-nine (39) strains (42%) were active against *E. coli* ATCC 25922. Six strains were active against *P. aeruginosa* ATCC 27853, while all strains tested were inactive against *E. aerogenes* ATCC 13048 as indicated with no or less than 50% growth inhibition. Twenty-three (23) active strains targeted 2–3 test pathogens, while 69 active strains were only active against one test pathogen (Supplementary Table S4).

### Phylogenetic diversity of multiple antibiotic-producing strains

The 92 active actinomycete strains were further identified and confirmed as *Streptomyces* species based on genomic analysis of their 16S rRNA and *rpoB* gene sequences. Comparison of 16S rRNA gene sequences (ranging from 1150 to 1500 nucleotides) and *rpoB* (700–995 nucleotides) gene sequences with their similar matches in the GenBank verified that the 92 active strains were closely related (97 to 100%) with 19 species under the genus *Streptomyces*. The nearly complete 16S rRNA and *rpoB* gene sequences were analyzed in a phylogenetic tree using maximum likelihood algorithms. The 16S rRNA and *rpoB* gene sequences of active strains reported in the present study were deposited in the GenBank nucleotide database (Supplementary Table S5).

Phylogenetic analysis revealed multiple strains with identical 16S rRNA gene sequences which clustered together into 13 major clusters (shown by the colored nodes in the tree) with high bootstrap values (> 90%) in the phylogenetic tree (Supplementary Fig. S3). Thirty-three strains (36%) were highly similar to *S. parvulus* presented in red circle. Followed by 12 strains with high similarity to S*. enissocaesilis* (light blue circle), 11 *S. rochei* strains (dark pink), six *S. mutabilis* strains (dark blue), five *S. diastaticus* strains (light green), four *S. kunmingensis* strains (green) and three *S. geysiriensis* strains (light orange). A phylogenetic analysis of *rpoB* gene sequence was conducted to provide a better resolution of the evolutionary relationship among strains within and between species supporting the taxonomic identity of the phylogenetically identical strains.

**Figure 2:**
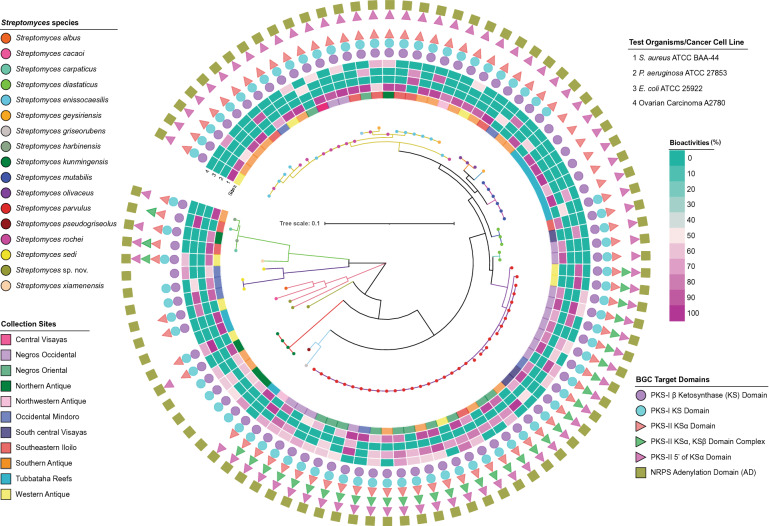
The phylogenetic tree of antibiotic-producing *Streptomyces* according to *rpoB* gene sequences. The different species were represented by colored circles in the tree. Clusters indicated by colored nodes in the tree were selected based on ≥ 90% bootstrap replicates. The collection sites of strains were annotated by the color strips adjacent to the tree. Heatmap with color gradient ranging from blue-green (no activity) to purple (100% activity) corresponds to the bioactivities of strains. Detected BGCs using PKS and NRPS primer sets were represented by colored shapes next to the heatmap. The tree was generated with maximum-likelihood algorithms using mega 7.0 (1000 bootstrap replications) and the substitution model Tamura–Nei method. The tree was drawn to scale, with branch lengths in the same units as those of the evolutionary distances used to infer the phylogenetic tree. The tree was visualized and annotated using iTOL 5.6.3 (Biobyte solutions, Heidelberg, Germany).

Similarly, the *rpoB* gene sequences phylogenetic tree (Fig. 2) showed 13 major clusters that were highly supported with bootstrap replicates > 90%, except for monophyletic clusters III (*Streptomyces* sp. strain DSD176) and X (*Streptomyces* sp. strain DSD1006) with low bootstrap replicates (< 90%). Active strains under cluster II and III were resolved *S. diastaticus* and *S. geysiriensis* lineages, respectively. *Streptomyces* sp. strain DSD176 under cluster II showed high similarity with *S. geysiriensis* based on its 16S rRNA gene sequences but separated from the other active *S. geysiriensis* strains found under cluster VII based on their *rpoB* gene sequences. Meanwhile, two active strains with high bootstrap support (98%) under cluster II diverged from the remaining active *S. diastaticus* strains in cluster VI, where cluster II being a sub-phyletic sister clade of active strains under cluster VI with similar 16S rRNA gene sequence matches in the GenBank. Interestingly, cluster VII was supported by 95% bootstrap replicates and comprised of sub-phyletic lines of 25 active strains belonging to three *Streptomyces* species: *S. enissocaesilis* (12 strains), *S. rochei* (11 strains), *S. geysiriensis* (two strains). *Streptomyces parvulus* (33 strains) and both *S. enissocaesilis* (12 strains), and *S. rochei* (11 strains) were the most dominant among the 19 bioactive *Streptomyces* species with the highest number of active strains. Interestingly, from the 92 active strains, we identified three novel species prospects: *Streptomyces* sp. strain DSD1006 from Northwestern Antique, *Streptomyces* sp. strain DSD3025 from Tubbataha Reefs and *Streptomyces* sp. strain DSD742 from Western Antique with 97–98% similarity with their reference match strains.

### Antibiotic and anticancer variations within phylogenetic clusters

We observed varying antibiotic and anticancer strengths exhibited by multiple strains despite their similar taxonomic identities (Fig. 2). In 33 active strains that were closely related to *Streptomyces parvulus*, there were nine strains with more than 90% growth inhibition against a multidrug-resistant strain of *S. aureus*. The remaining 24 active *S. parvulus* strains have moderate antibacterial activities ranging from 50 to 89% growth inhibitions against *S. aureus* ATCC BAA-44, *E. coli* ATCC 25922 and *P. aeruginosa* ATCC 27853. Only 19 isolates have exhibited moderate cytotoxic activity ranging from 50 to 70% to ovarian carcinoma (A2780) (Supplementary Table S4). The antibacterial and anticancer activities in terms of % growth inhibition and the number of test pathogens inhibited by *S. parvulus* strains were shown in the outer ring heat map of phylogenetic tree. These variations in antibacterial and anticancer activities were also evident intraspecies found in other clusters in the phylogenetic tree.

**Figure 3:**
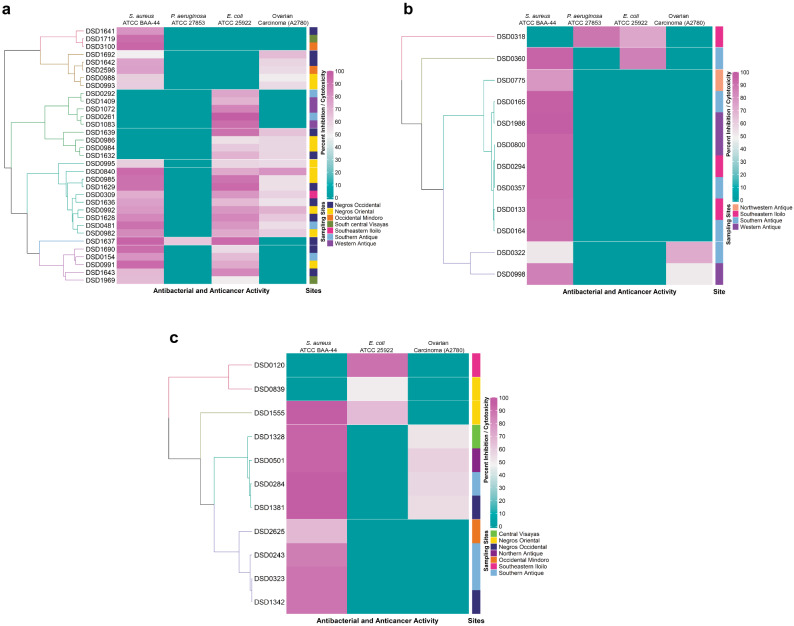
Heat Map based on the antibacterial and anticancer activity of the three dominant bioactive *Streptomyces* species, (**a**) *S. parvulus*, (**b**) *S. enissocaesilis*, and (**c**) *S. rochei,* against four test pathogens and ovarian cancer cell line (A2780). Dendrogram on the left of the diagram indicate the relatedness of the strains based on their bioactivities. Color gradient ranging from blue-green (0%), pink (50%), and purple (100%) corresponds to the antibacterial and cytotoxicity activity. Collection sites of strains were annotated by the color strips on the right-side tree of the diagram. The heat map was created using RStudio statistical program ver1.2.5042 with heat map clustering methods; hierarchical clustering Euclidean distance metric was used to cluster the data.

*Streptomyces* strains nearly identical to *S. rochei* (11 strains), *S. ennisocaesilis* (12 strains) and *S. geysiriensis* (three strains) (Supplementary Fig. S3) revealed ambiguous phylogenetic affiliation. These intraspecies morphotypes have varying and remarkable antibacterial activities, as demonstrated in the purple-green portion of the tree and the outer ring heat map in Fig. 2. Furthermore, the intensity of antibacterial and anticancer activities and the intraspecific diversity of three dominant active *Streptomyces* species isolated in the west-central Philippines was presented in heat map clusters (Fig. 3a–c). In the heat map of *S. parvulus* active strains (Fig. 3a), seven different clusters were formed according to bioactivity against the test pathogens and ovarian carcinoma. The largest cluster formed nine strains was active against *S. aureus* ATCC BAA-44 and *E. coli* ATCC 25922 and all have cytotoxic activity ranging from 50 to 70% against ovarian carcinoma. Majority of the strains from this cluster were isolated from two neighboring sites, Negros Occidental and Negros Oriental (Fig. 1a). *Streptomyces enissocaesilis* (Fig. 3b) formed four clusters: the largest cluster composed of eight strains with antibiotic activity ranging from 80 to 100% growth inhibition against *S. aureus* ATCC BAA-44. Interestingly, no isolates from this cluster showed cytotoxic activity against ovarian carcinoma. Lastly, *S. rochei* strains (Fig. 3c) formed four different clusters. Two clusters formed, with four isolates each, were active against *S. aureus* ATCC BAA-44. One cluster, composed of DSD284, DSD1328, DSD501, and DSD1381 exhibited cytotoxic activity. The *S. rochei* strains from Northern Antique and Central Visayas are all found in this cluster. The differences in antibiotic and anticancer activities shown in this study demonstrate intraspecies variations within multiple strains despite they share nearly identical 16S rRNA gene and *rpoB* sequences. Intraspecies variations in antibiotic and anticancer activities were observed in *S. diastaticus*, *S. kunmingensis*, and *S. mutabilis* active strains (Supplementary Fig. S4A–C).

### PCR-based detection of PKS and NRPS gene domains

We explored the PCR-based BGC profile and the extent of BGC diversity in multiple strains within and between species. The occurrence of NRPS, type-I and type-II PKS biosynthetic genes were assessed through PCR amplification approach^37–41^. Visualization of strong unambiguous amplicons was shown in Supplementary Fig. S5–7. One primer set (A3F, A7R) was used to amplify adenylation (AD) domains of NRPS genes, while different primer sets were used to detect PKS type-I (KSMA-F, KSMB-R; KS-F, KS-R) and PKS type-II (KS1-F, KS1-F; KSα, KSβ; 540F, 1100R) genes in this study. Amplification homology of PKS domains by several primer sets used in this study may suggest occurrence of additional PKS gene clusters in the bioactive strains. The detection of amplified genes by the primers used in this study signified the biosynthetic potential of the bioactive strains and increased the likelihood to produce polyketides and non-ribosomal peptides with antibacterial and anticancer properties. The lack of amplification of ketosynthase (KS) and adenylation (AD) gene fragments may indicate absence of PKS and NRPS systems, or the BGC machineries responsible for the bioactivities of antibiotic-producing *Streptomyces* species have lower homology with the primers used.

As illustrated in Fig. 2, all 19 *Streptomyces* species have NRPS gene based on the positive amplification of the adenylation domain. However, we found variation in PKS type-I and -II genes between *Streptomyces* species. Majority of the species harbor both target regions in PKS I gene. Only *Streptomyces* sp. strain DSD3025 did not harbor the PKS-I gene. Strains identical to *Streptomyces mutabilis* do not have the KS domain conserved region targeted by KS-F and KS-R primers (Supplementary Fig. S6). Interspecies diversity was evident in the domain content of PKS-II as well. Interestingly, only four species, *S. parvulus*, *S. griseorubens*, *S. carpaticus* and *S. xiamenensis*, carried KSα and KSβ domain complex of PKS II at 500–600 bp, which is contrary to the expected 800–900 expected length of target region^39^ (Supplementary Fig. S7B.1–B.2). This amplification of KSα and KSβ domain complex can be associated to the complexity and diversity of PKS type-II compounds. Strains with nearly identical 16S rRNA and *rpoB* gene sequences to *S. kunmingensis*, *S. sedi*, and *Streptomyces sp*. strain DSD3025 did not harbor all target domains of PKS II in their genomes. The PKS diversity may infer differences in the metabolite production, explaining the antibiotic activity variation observed between species. Multiple strain comparative analysis clearly showed that taxonomically related strains shared similar set of NRPS and PKS domain contents. Furthermore, identical strains isolated from geographically distant locations still harbored uniform NRPS and PKS domain contents.

Interestingly, we also observed variation in the amplification of PKS type I and type II genes within the strains of *Streptomyces sedi* and *Streptomyces carpaticus* (Fig. 2). The *S. sedi* strain DSD3018 showed the absence of PKS-1 gene KS domain region targeted by the KS-F and KS-R primers which is contrary to the other *S. sedi* strains, DSD3011 and DSD2987. *Streptomyces carpaticus* strain DSD331 harbors the 5’ portion of KSα domain of PKS-II gene; however, absence of this sequence was observed in *S. carpaticus* strain DSD274. These variations may suggest the presence of sequence variants in the same region of KS and KSα domains.

### Isolation technique and carbon source utilization profiles

**Figure 4:**
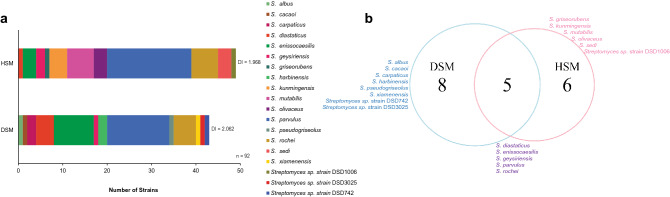
Diversity of antibiotic-producing *Streptomyces* using culture-dependent isolation techniques, heat shock method (HSM) and dry stamp method (DSM)**.** (**a**) Bar graph showed the number of active strains cultivated by the two culture-dependent techniques where heat shock method (HSM) produced higher number of active strains, but dry stamp method (DSM) yielded more diverse strains. (**b**) Venn diagram of two isolation techniques showed that five *Streptomyces* species were isolated using both techniques.

Culture-dependent techniques and carbon source composition of minimal marine media enhanced the isolation of active marine sediment-derived *Streptomyces* strains. We found that the heat-shock method (HSM) yielded higher recovery of active actinomycete strains (49 strains, 53%) as compared to dry stamp method (DSM) (43 strains, 47%) (Fig. 4a). However, active *Streptomyces* species were more diverse when processed using DSM (diversity index = 2.062) as compared to HSM (diversity index = 1.968).

The Venn diagram (Fig. 4b) showed that the 5 dominant species (> 1% abundance) can be recovered in both methods. We also noted that some species were exclusively recovered using a specific method. Eight species were exclusively recovered in DSM compared to 6 species in HSM. All active strains of *S. kunmingensis, S. mutabilis, S. sedi* and *S. olivaceus* were only recovered by HSM. Contrary, the active strains of *S. carpaticus* and *S. harbinensis* were only isolated using DSM.

**Figure 5:**
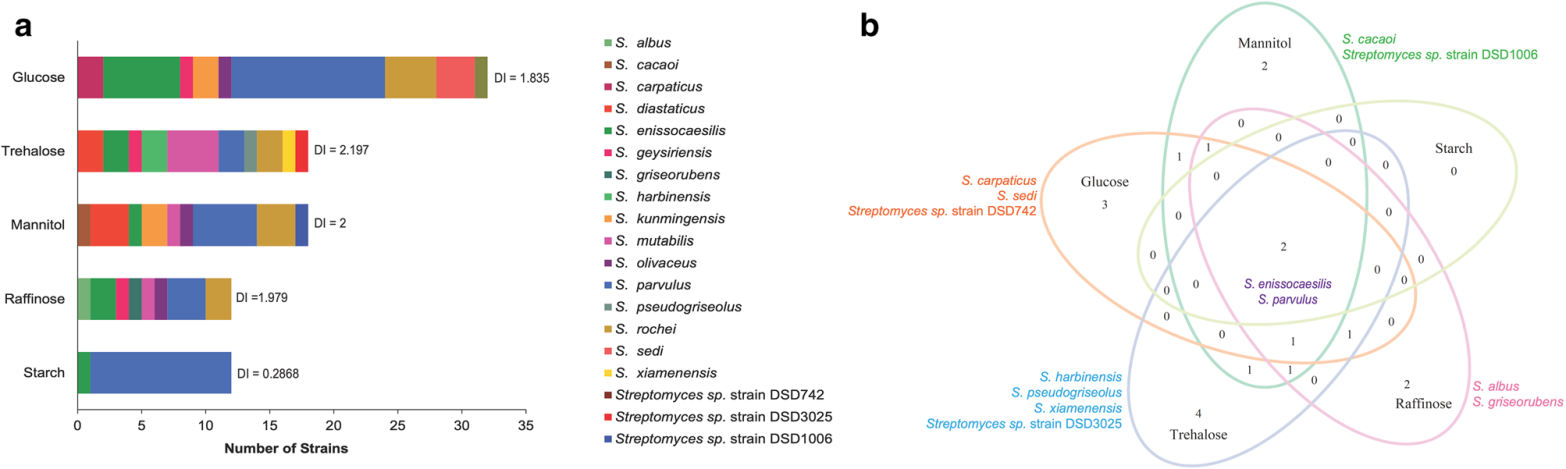
Diversity of antibiotic-producing *Streptomyces* using five different carbon sources. (**a**) From the five carbon sources in the minimal marine media utilized by *Streptomyces* strains in this study, mannitol yielded the highest number of active strains, while high diversity was recorded in active strains that utilized glucose (n = 92). (**b**) Venn diagram of five carbon sources showed that two *Streptomyces* species can be isolated using all five carbon sources.

Carbon source composition of marine minimal media, along with effective isolation techniques, was crucial for the isolation of antibiotic-producing *Streptomyces*. Among the five minimal marine media used, three carbon sources yielded high isolation rate: glucose, mannitol, and trehalose yielded nine species with 32 strains (35%), nine species with 18 strains (20%), and ten species with 18 strains (20%) respectively. However, only eight species (12 strains, 20%) and two species (12 strains, 20%) were isolated in raffinose, and starch-based media, respectively. In the contrary, high diversity indices were observed in trehalose (2.197), mannitol (2.0), and raffinose (1.979) (Fig. 5a). As expected, the starch-containing media had the lowest isolation rate and diversity as only two species (*S. enissocaesilis* and *S. parvulus*) were able to utilize a more complex carbon source.

Interestingly, co-isolation of species in different carbon sources was shown in the Venn diagram (Fig. 5b). Eight species can be recovered from at least two different carbon sources, whereas 11 species were exclusively isolated from a specific carbon source. Bioactive *S. enissocaesilis* and *S. parvulus* strains were recovered from all of the carbon sources utilized in this study. Active *S. rochei* were isolated in four media but not in starch-based media. In contrast, more exclusive species were isolated in trehalose with four species (*S. harbinensis* strains, *Streptomyces* sp. strain DSD3025, *S. pseudogriseolus*, and *S. xiamenensis*). Followed by glucose with three species (*Streptomyces* sp. strain DSD742, *S. carpaticus* and *S. sedi*), and two species each on mannitol- and raffinose-based media (Fig. 5b). The results indicated that diverse *Streptomyces* species preferred simple sugars-containing one or two sugar molecule as nutrient source compared to complex sugars.

### *Streptomyces* abundance and diversity in geographical sampling locations

**Figure 6:**
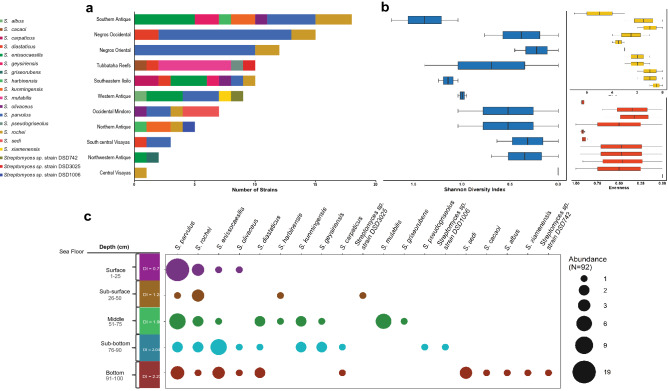
(**a**) The abundance profile of 19 antibiotic-producing *Streptomyces* species in different geographical location across the Philippines revealed that *S. parvulus* was the most abundant species. (**b**) Diversity, richness and evenness of antibiotic-producing *Streptomyces* species in different sites were calculated using vegan package in RStudio ver1.2.5042 (https://www.rstudio.com/). (**c**) Recovery profile and abundance of antibiotic-producing strain per depth layer, indicated by colored circles and its size, revealed that strains in the bottom sediments were the most diverse as compared to other depth layer and *S. parvulus* was the most abundant species recovered.

Bioactive *Streptomyces* species were widely distributed across the different sampling locations in west-central Philippines (Fig. 6a). Although Southern Antique, Negros Occidental and Negros Oriental have highest number of active strains isolated, we found that Southern Antique, Southern Iloilo, and Western Antique were the most diverse sampling sites (Fig. 6b). We have isolated the greatest number of antibiotic-producing *Streptomyces* species which were evenly distributed in Southern Antique. This indicates that Southern Antique is stable with many potential niches that can support highly diverse S*treptomyces* species.

*Streptomyces parvulus* emerged as the dominant antibiotic-producing species in this study. Out of the 33 active *S. parvulus* strains, the majority were isolated in Negros Occidental (11 strains) and Negros Oriental (ten strains). Although Negros Occidental and Negros Oriental have high abundance, its microbial community is highly dominated by one species, *S. parvulus*, supported by the low species richness, evenness, and diversity (Fig. 6b). This finding implies that these sites may have few potential niches that only a few species dominate. Bioactive *S. enissocaesilis* strains were recovered in four sampling locations only; specifically, Southern Antique (five strains), Western Antique (three strains), Southeastern Iloilo (three strains), and Northwestern Antique (one strain). Active *S. rochei* were isolated and evenly distributed in seven sampling locations, but were not present in Western Antique, Northwestern Antique, South Central Visayas, and Tubbataha Reefs. Notably, we observed that no bioactive *S. parvulus*, *S. enissocaesilis,* and *S. rochei* were isolated in Tubbataha Reefs, but antibiotic-producing *S. cacaoi, S. psuedogriseolus* and *S. mutabilis* strains were isolated only in Tubbataha Reefs marine sediments. Meanwhile, site-specific species such as *S. sedi* were recovered only in Occidental Mindoro. The isolation of site-specific species within genus *Streptomyces* can offer insight on the adaptive capacity of strains to inhibit locally coexisting resource competitors within and among these distinct locations.

### Distribution of bioactive *Streptomyces* species at different sediment depths

We further investigated the distribution of antibiotic-producing *Streptomyces* strains along with the 110-cm sediment depth in different sampling sites. From the sediments that were partitioned according to depth with five categories at 25-cm increments, heterogeneous distributions of bioactive species were observed at deeper sediment with different dominant species in each depth (Fig. 6c). Although, *S. parvulus*, *S. rochei*, and *S. enissocaesilis* strains were ubiquitous in all depths, several species thrive abundantly in specific depths compared to other species. *Streptomyces parvulus* was the most dominant species in surface sediment. Meanwhile, *Streptomyces rochei* and *S. enissocaesilis* strains were more adapted in surface and sub-bottom sediments, respectively. Depth-specific *Streptomyces* strains were also identified as follows: *Streptomyces* sp. strain DSD3025 was isolated in subsurface sediments; *S. mutabilis* strains were abundant in the middle sediment layer; *Streptomyces sp*. strain DSD1006 and *S. pseudogriseolus* strain were recovered from sub-bottom sediments; and *Streptomyces* strain sp. DSD742, *S. albus*, *S. sedi* and *S. xiamenensis* strains were obtained from bottom sediments. High species diversity was positively correlated with increasing sediment depth, where surface sediments are known to be more prone to dispersal and wash-offs by environmental factors such as deep ocean currents^42^. Furthermore, the depth-specific species identified largely influenced the species richness in varying sediment depth.

## Discussion

The stimulation of antibiotic research and development from new and emerging resources has a pivotal role in developing strategies to address antibiotic resistance's global health threat^43^. Members of the genus *Streptomyces* remain as the powerhouse of structurally diverse antibiotics^4–7^. In the tropics, most antibiotic-producing actinomycete studies were focus on terrestrial soil^44, 45^, mangrove communities^46^ and marine sediments^23, 24^. The Philippine archipelago lies within the Coral Triangle or the world's center for marine biodiversity and is considered as a biogeographic realm of tropical oceanogeographic features that provide different marine ecosystems for diverse marine macrofauna and microbial community^47^. In recent report, actinomycete was found to be a dominant phylum in seawater samples collected in Benham Rise, also known as Philippine Rise^23^.

Here, we focused on marine sediments as an ecological niche, where we collected marine sediment samples from 11 untapped sampling sites across the Philippine archipelago to isolate *Streptomyces* strains and unveil their bioactivity, phylogeny, diversity, abundance, and distribution. Marine sediment samples were processed by two culture-dependent techniques designed to eliminate undesirable growth of other bacteria and maximize cultivatable actinomycetes' isolation, specifically *Streptomyces*. Isolation of actinomycete strains results from an effective combination of culture-dependent techniques and selective carbon source utilization, despite the competition for space and resources under laboratory conditions. Successful isolation of actinomycete strains enabled us to overcome the mimicking problem of essential environmental needs, *e.g.,* culture medium and seawater composition, to cultivate actinomycetes in the laboratory. Thus, the large-scale isolation of 2212 actinomycete strains from Philippine marine sediments allowed us to understand the geographical distribution and recovery profile of antibiotic-producing actinomycetes in Philippine archipelago. The highest recoveries of actinomycete strains were recorded in Negros Occidental, Southern Antique, and the Southeastern Iloilo. Interestingly, these sampling sites lie within the 600 × 400 km^2^ area of the semi-closed tropical Sulu Sea^48^, characterized by quasi-steady water circulation and bottom topography (Fig. 1)^49^.

To delineate Philippine marine sediment-derived actinomycetes' capability as antibiotic metabolite producer, qualitative and quantitative approaches were utilized as antibacterial screening strategies. The majority of the strains confirmed antibacterial activities against *S. aureus* ATCC BAA-44, an Iberian clone of Methicillin-resistant *S. aureus* (MRSA) with known resistance against 18 commercially available antibiotics^26, 27^. Meanwhile, despite expected low or moderate susceptibility of Gram-negative test pathogens^50^, active *Streptomyces* strains from Philippine marine sediments have shown promising activity against this pathogen.

The phylogenomic analysis of 92 antibiotic-producing strains resulted in identifying 13 major *Streptomyces* clusters, including three strains that may represent novel species with 97–98% 16S rRNA gene sequence similarity with reference strain in the GenBank. The three novel species identified were isolated from our sampling site in the Sulu Sea. One novel species were isolated in Tubbataha Reefs, and two sites were facing the Sulu Sea (Western Antique and Northern Antique). Considering that most secondary metabolite-producing *Streptomyces* are hardly culturable under laboratory conditions, the isolation of active strains in the Philippines using culture-dependent approaches is promising and far from exhausted^51^. The interspecies diversity of active *Streptomyces* recovered from different Philippines sampling sites reflects the current condition of identified sampling sites, where high diversity and abundance of microbial communities were observed in these underexplored and less exploited areas. Although Negros Occidental has the highest number of recovered actinomycete strains, the most diverse antibiotic-producing *Streptomyces* were isolated from Southern Antique.

The high *Streptomyces* recovery of active strains by heat shock method (HSM) revealed that exposure to relatively high temperature (45–55 °C) enhanced active *Streptomyces'* isolation. However, completely air-dried marine sediment samples pressed directly in the culture medium (DSM) yielded more diverse active strains. Carbohydrate utilization is crucial in isolating *Streptomyces* from marine sediments and may vary between species. The highest diversity of active strains was observed on a culture medium containing glucose, a rapidly assimilated carbon source. It is noted, however, that glucose was associated with suppressing and interfering with the formation of many antibiotics^52,53^, and sometimes represses the catabolism of glucose itself^53^. Thus, the depletion of carbon and other nutrient sources is resolved by growing the actinomycetes strains in MM1, an enriched marine medium. Starch is the slowly utilized carbon source component of MM1 that may trigger idiolite production in actinomycete strains^54^.

Microorganisms under the genus *Streptomyces* are ubiquitous in the environment and play essential roles in terrestrial ecosystems, especially in nutrient cycling and soil substrate decomposition^55^. In this study, we were able to identify ubiquitous antibiotic-producing *Streptomyces* strains, highlighting strains with 16S rRNA and *rpoB* gene sequence analysis identical with *S. parvulus* found in the largest cluster of the phylogenetic tree. The ubiquity of species under genus *Streptomyces* suggests their secondary metabolite arsenal's evolutionary success that can be correlated with their ecological functions such as breakdown of plant biomass and nutrient cycling^55, 56^. Furthermore, the role of symbiosis and *Streptomyces*' adaptive capability is crucial, particularly in their gene machineries for the biosynthesis of secondary metabolites^57^. The successful cultivation of *Streptomyces* strains in the laboratory as mediated by optimizations of medium and culture-dependent conditions can lead to more than one bioactive compound with antibacterial activities^58^. Secondary metabolites from unexplored gene clusters of ubiquitous *Streptomyces* species can drive the discovery of novel lead compounds with extensive applications^59^.

The dominant antibiotic-producing strains identified in this study were the common *Streptomyces* species in the one-meter depth of sediments collected in different geographical locations. Upon tracing the abundance of active strains across depths, we observed that the dominant and active *Streptomyces* species identified decreases gradually in number with increasing sediment depth. Stratification in microbial community composition occurred in parallel to drops in microbial activity and abundance caused by reduced energy availability below the mixed sediment surface layer^60^. Depth-specific bioactive strains and their corresponding species identities signify that microbes are major drivers of diversification and may shape the microbial communities in different sediment horizon layer^61^. Our genomic and phylogenetic analyses have identified a distinct assemblage of diverse *Streptomyces* lineages occurring from 50 to 110 cm-depth below the seafloor, representing a significant portion of antibiotic-producing *Streptomyces* community at different geographical locations. Enriched *Streptomyces* species composition was evident at increasing sediment depth horizons associated with adaptive and high fitness capacity of active strains in deeper marine sediment horizons. In contrast, in a study that focused on the benthic foraminifera from multiple-core samples collected in the Sulu Sea basin, diversity and faunal abundances of these benthic organisms decreased with increasing water depth^62^. The recent surfacing of *Streptomyces* isolation and profiling may provide insights in investigating *Streptomyces* species that could be isolated from different geographical sediment depth horizons in the Philippines.

Sole investigation of *Streptomyces* bioactivities without correlation to secondary metabolites and BGC production is not beneficial for discovering potential new antibiotics. The different sets of primers in detecting BGCs for polyketide and non-ribosomal peptides can generate structural diversity in terms of possible fully assembled compounds produced by active *Streptomyces* strains. The KS domains were targeted for PKS type-I detection as they are highly conserved and tend to cluster phylogenetically based on the secondary metabolites they produce^37^. Since PKS type I genes encode more than one PKS gene cluster, PCR products obtained with the KS-specific primers may represent mixtures of the KS-coding sequences^63^. We observed that strains closely related to *Streptomyces parvulus*, *S. carpaticus, S. griseorubens*, and *S. xiamenensis* have the only positive results in type II polyketide targeting the KSα KSβ domain complex used in this study. Ketosynthase (KS or KSα) domains are common PKS-II machineries. Still, the functional synergy of KSα and the chain length factor (CLF or KSβ) enabled *Streptomyces* species to produce PKS-II polyketides differing in length and cyclization. Detection for NRPS biosynthetic gene pathways revealed that all 92 active *Streptomyces* strains have NRPS genes. Notably, taxonomically closely related strains belonging to the same *Streptomyces* species have similar biosynthetic pathways responsible for their antibacterial activities.

Intraspecies variation on the KS domains amplification observed on *S. sedi* and *S. carpaticus* active strains using PKS primer sets (KSF, KSR; 540F, 1100R) may suggest sequence variants in similar regions of KS and KSα domains. In a recent study on the diversity of PKS type-I gene, they identified two amino acid sequence variants in the same region of PKS-I KS domain, EA_(C)_HGTGT or EAHATST. EA_(C)_HGTGT is commonly found in highly reducing-type PKSs, while EAHATST is common for partially reducing-type PKSs (*e.g*., MSAS)^64^. Sequence variants in the same domain region of active strains could have hindered the amplification of the targeted regions of primers used in this study.

Despite similarities in 16S rRNA and *rpoB* gene-based analysis, multiple strains of the same *Streptomyces* species from Philippine sampling sites differed in their intensity of inhibiting the growth of clinically relevant pathogens such as multidrug-resistant *S. aureus* and *E. coli* and *P. aeruginosa*. Sampling site-specific strains are composed primarily of mobile genetic elements and genes that are likely acquired by horizontal gene transfer^13^. The varying range of antibacterial activities of intra- and interspecies *Streptomyces* strains and restrictions of strains to specific sampling sites can be attributed to *Streptomyces'* adaptive characteristics outcompete other species in marine sediment microbial community.

The isolation of marine sediment-derived actinomycetes with promising antibiotic activities from different geographical sites in the west-central Philippines signifies the need to explore remote areas in the tropics and urge to preserve the marine environment in these locations away from increasing anthropogenic activities. This study further highlights the importance of sampling multiple taxonomically identical strains in improving our efforts in bioprospection. New antibiotics with novel inhibitory mechanisms are urgently needed as multidrug-resistant pathogens are rapidly evolving, affecting human, animal, and environmental sectors. Dereplication strategies usually discard strains of known species as they may produce already known metabolites. However, our study has pointed out that strains of even known species may serve as producers of unique antibiotics against clinically relevant pathogens, thus offering new chemical space for antibiotic discovery.

## Conclusions

In summary, we have successfully recovered *Streptomyces* species from marine sediments across the Philippines with three possible novel species (*Streptomyces* sp. strains DSD742, DSD1006, and DSD3025) in our collection. Although widely distributed, *Streptomyces* were diverse and abundant in untapped preserved environments across the Philippine archipelago. Aside from its diversity, the bioactivity of all isolated *Streptomyces* species was assessed, revealing that multiple species at strain-level exhibit differences in the degree of their bioactivity. Detection of the BGCs of antibiotic-producing *Streptomyces* species showed that closely related strains share similar biosynthetic genes to produce secondary metabolites. Although this is true, not all species at strain-level share this similarity, indicating that different strains of the same species can produce chemically diverse compounds. The variations in the antibacterial and anticancer activity at strain-level and the presence of BGCs on *Streptomyces* species provide opportunities to explore the biosynthetic capability of this microbe for the discovery of natural compounds. Thus, future metabolomics analysis of the biomass warrants the profiling and identification of the natural products.

Further work using genomic approaches such as the sequencing of the detected BGCs (PKS and NRPS) on mining of the biosynthetic genes and metabolomic studies using liquid chromatography mass spectrometry (LCMS) in analyzing secondary metabolites produced by the *Streptomyces* will lead us to fully understand what these inter-and intraspecies can produce for the detection and isolation of novel natural compounds. Results obtained in this study uncover the richness of the natural resources in the Philippines. As part of the Coral Triangle with diverse marine habitats, marine sediments in the Philippines have proven as an important ecological niche in isolating microorganisms such as the *Streptomyces* with antibiotic and anticancer activities.

## Supplementary Information

Supplementary Information.
