# Publisher Correction: Multiple strain analysis of *Streptomyces* species from Philippine marine sediments reveals intraspecies heterogeneity in antibiotic activities

**DOI:** 10.1038/s41598-021-98601-9

**Published:** 2021-09-20

**Authors:** Chuckcris P. Tenebro, Dana Joanne Von L. Trono, Carmela Vannette B. Vicera, Edna M. Sabido, Jovito A. Ysulat, Aaron Joseph M. Macaspac, Kimberly A. Tampus, Trisha Alexis P. Fabrigar, Jonel P. Saludes, Doralyn S. Dalisay

**Affiliations:** 1 Center for Chemical Biology and Biotechnology (C2B2), University of San Agustin, 5000 Iloilo City, Philippines; 2 Center for Natural Drug Discovery and Development (CND3), University of San Agustin, 5000 Iloilo City, Philippines; 3 Department of Chemistry, College of Liberal Arts, Sciences, and Education, University of San Agustin, 5000 Iloilo City, Philippines; 4 Department of Biology, College of Liberal Arts, Sciences, and Education, University of San Agustin, 5000 Iloilo City, Philippines; 5 Balik Scientist Program, Department of Science and Technology, Philippine Council for Health Research and Development (PCHRD), 1631 Bicutan, Taguig City, Philippines

Correction to: *Scientific Reports* 10.1038/s41598-021-96886-4, published online 02 September 2021

The original version of this Article contained an error in Figure 5, where panel b did not display correctly. The original Figure 5 and accompanying legend appear below.

**Figure 5:**
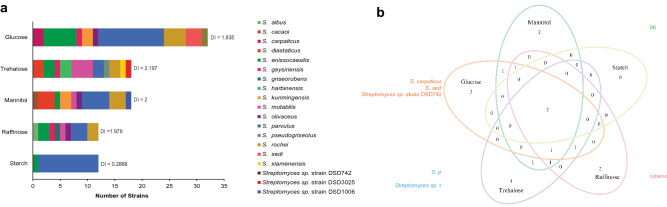
Diversity of antibiotic-producing *Streptomyces* using five different carbon sources. (**a**) From the five carbon sources in the minimal marine media utilized by *Streptomyces* strains in this study, mannitol yielded the highest number of active strains, while high diversity was recorded in active strains that utilized glucose (n = 92). (**b**) Venn diagram of five carbon sources showed that two *Streptomyces* species can be isolated using all five carbon sources.

The original Article has been corrected.

