# Supplementary material for: Multiple strain analysis of Streptomyces species from Philippine marine sediments reveals intraspecies heterogeneity in antibiotic activities
Source: Sci Rep. 2021 Sep 2;11:17544. doi: 10.1038/s41598-021-96886-4 (PMC8413401; doi:10.1038/s41598-021-96886-4)
Supplement: Supplementary file 1 — Supplementary Information. [file 41598_2021_96886_MOESM1_ESM.pdf]

Multiple Strain Analysis of *Streptomyces species* from Philippine Marine Sediments Reveals Intraspecies Heterogeneity in Antibiotic Activities

Chuckcris P. Tenebro[1], Dana Joanne Von L. Trono[1], Carmela Vannette B. Vicera[2], Edna M. Sabido[2], Jovito A. Ysulat, Jr.[1], Aaron Joseph M. Macaspac[1], Kimberly Tampus[1], Trisha Alexis Fabrigar[1], Jonel P. Saludes[2,3,5] and Doralyn S. Dalisay[1,4,5,*]

[1] Center for Chemical Biology and Biotechnology (C2B2), University of San Agustin Iloilo City 5000, Philippines
[2] Center for Natural Drug Discovery and Development (CND3), University of San Agustin, Iloilo City 5000, Philippines
[3] Department of Chemistry, College of Liberal Arts, Sciences, and Education, University of San Agustin, Iloilo City 5000, Philippines
[4] Department of Biology, College of Liberal Arts, Sciences, and Education, University of San Agustin, Iloilo City 5000, Philippines
[5] Balik Scientist Program, Philippine Council for Health Research and Development (PCHRD), Department of Science and Technology, Bicutan, Taguig City, 1631, Philippines
* Correspondence: ddalisay@usa.edu.ph Tel. no. +63-33-5010350

SUPPLEMENTAL INFORMATION

Supplementary Table S1. Degenerate Primers Used to Amplify Polyketide Synthase (PKS) and Non-Ribosomal Peptide Synthetase (NRPS) Domain Fragments.

Entry	Target Gene for Amplification	Predicted Length (bp)	Primer Name	Primer Sequence (5' to 3')	References
1	Type I polyketide synthase β-ketoacyl synthase (KS) domains fragments	670	KS-F	CCS CAG SAG CGC STS YTS CTS GA	Ginolhac *et al.*, 2005
			KS-R	GTS CCS GTS CCG TGS GYS TCS A	
2	Type I polyketide synthase KS domain fragments	700	KSMA-F	TSG CSA TGG ACC CSC AGC AG	Ginolhac *et al.*, 2005
			KSMB-R	CCS GTS CCG TGS GCC TCS AC	
3	Type II polyketide synthases KSα and KSβ domain fragments	800-900	KSα	TSG RCT ACR TCA ACG GSC ACG G	Al-Amoudi *et al.*, 2016
			KSβ	TAC SAG TCS WTC GCC TGG TTC	
4	Type II polyketide synthases KSα domain fragments	613	KS1-F	TSG CST GCT TGG AYG CSA TC	Zothanpuia *et al.*, 2016
			KS1-R	TGG AAN CCG CCG AAB CCT CT	
5	Type II polyketide synthases at 5' portion of KSα genes	554	540F	GGI TGC ACS TCI GGI MTS GAC	Wawrik *et al.*, 2005
			1100R	CCG ATS GCI CCS AGI GAG TG	
6	NRPS Adenylation domain (AD) fragments	700	A3F	GCS TAC SYS ATS TAC ACS TCS GG	Ayuso-Sacido *et al.*,2005
			A7R	SAS GTC VCC SGT SCG GTA S	

Ginolhac, A., Jarrin, C., Robe, P., Perrière, G., Vogel, T. M., Simonet, P. & Nalin, R. Type I polyketide synthases may have evolved through horizontal gene transfer. *J. Mol. Evol.* **60**, 716-25 **(2005).**

Al-Amoudi, S., Essack, M., Simões, M. F., Bougouffa, S., Soloviev, I., Archer, J. A., Lafi, F. F. & Bajic, V. B., Bioprospecting Red Sea coastal ecosystems for culturable microorganisms and their antimicrobial potential. *Mar. Drugs.* **14**, 165 (**2016).**

Zothanpuia, Passari, A. K., Gupta, V. K. & Singh, B. P. Detection of antibiotic-resistant bacteria endowed with antimicrobial activity from a freshwater lake and their phylogenetic affiliation. *PeerJ* **4**, e2103 (2016).

Wawrik, B., Kerkhof, L., Zylstra, G. J. & Kukor, J. J. Identification of unique type ii polyketide synthase genes in soil. *Appl. Environ. Microbiol.* **71**, 2232 (2005).

Ayuso-Sacido, A.; Genilloud, O., New PCR Primers for the screening of NRPS and PKS-I systems in Actinomycetes: Detection and distribution of these biosynthetic gene sequences in major taxonomic groups. *Microb. Ecol.* **49**, 10-24 (2005).

Supplementary Table S2. Collection Sites of Marine Sediment Collection, Date of Collection and Coordinates.

Entry	Collection Site	Date of Collection	Coordinates
1	Southeastern Iloilo site 1	April, 2017	10.3302778 N,122.5875 E
	Southeastern Iloilo site 2	April, 2017	10.3275 N, 122.5969444 E
2	Southern Antique site 1	April, 2017	10.4191667 N, 121.9252778 E
	Southern Antique site 2	April, 2017	10.41472222 N, 121.9380556 E
3	Western Antique site 1	April, 2017	11.3188889 N, 121.6616667 E
	Western Antique site 2	April, 2017	11.3158333 N, 121.6775 E
4	Northern Antique	April, 2017	11.41722222 N, 122.0288889 E
5	Northwestern Antique	April, 2017	11.4763889 N, 121.9316667 E
6	Negros Occidental	May, 2017	9.87222222 N, 122.3769444 E
7	Negros Oriental	May, 2017	9.086218 N, 123.2688889 E
8	Central Visayas	May, 2017	9.43222222 N, 123.3891667 E
9	South Central Visayas	May, 2017	9.51666667 N, 123.7119444 E
10	Occidental Mindoro site 1	March, 2018	12.665 N, 120.4119444 E
	Occidental Mindoro site 2	March, 2018	12.65833333 N, 120.4130556 E
11	Tubbataha Reefs site 1	April, 2018	8.88861111 N, 119.9666667 E
	Tubbataha Reefs site 2	April, 2018	8.74 N, 119.8191667 E

Supplementary Table S3. Distance of Sampling Sites from the Mainland and Marine Sediment Sample Description.

Entry	Sampling Location	Distance from mainland (m)	Number of sediment cores sampled	Marine Sediments Characteristics
1	Southeastern Iloilo	1000	2	Coarse mixture of muddy sand and pebbles
2	Southern Antique	250	2	Fine to coarse grains of ash gray sand
3	Western Antique	700	2	White grains of sand and coral patches
4	Northern Antique	400	2	Fine white grains of sand, small pebbles, and tiny pieces of broken corals
5	Northwestern Antique	200	1	Fine white grains of sand, small pebbles, and tiny pieces of broken corals
6	Negros Occidental	350	1	Fine to slightly coarse mixture of white grains of sand and small pebbles
7	Negros Oriental	200	1	Fine to slightly coarse mixture of white grains of sand and small pebbles
8	Tubbataha Reefs	15000	2	Fine to coarse white sediments with red and white patches of corals
9	Central Visayas	200	1	Fine to coarse mixture of white grains of sand, small pebbles, and tiny pieces of broken corals
10	South Central Visayas	150	1	Fine to slightly coarse mixture of white grains of sand and small pebbles
11	Occidental Mindoro	300	2	Fine grains of white sand with pebbles and coral patches

Supplementary Table S4. Antibacterial and Anticancer Activity Profile of Active *Streptomyces* Strains

**Isolate Code and Positive Control**	**16S rRNA Gene Sequence Match (97 to 99% identity)**	**Collection Site**	**Antibacterial Activities % Growth Inhibition ± SD** (conc. of extract = 2.5 mg/ml)				**Anticancer Activity % cytotoxic activity ± SD** (conc. of extract = 400 µg/ml)
			S. aureus ATCC BAA-44	*E. coli* ATCC 25922	*P. aeruginosa* ATCC 27853	*E. aerogenes* ATCC 13048	Ovarian Cancer (A2780)
Streptomyces sp. strain DSD1715	*Streptomyces albus*	Western Antique	---	80 ± 0.06	---	---	---
Streptomyces sp. strain DSD2595	*Streptomyces cacaoi*	Tubbataha Reefs	95 ± 0.47	---	---	---	---
Streptomyces sp. strain DSD331	*Streptomyces carpaticus*	Southeastern Iloilo	92 ± 0.50	---	---	---	---
Streptomyces sp. strain DSD274	*Streptomyces carpaticus*	Southeastern Iloilo	---	96 ± 0.19	---	---	---
Streptomyces sp. strain DSD299	*Streptomyces diastaticus*	Southeastern Iloilo	81 ± 0.51	---	---	---	---
Streptomyces sp. strain DSD1886	*Streptomyces diastaticus*	Negros Occidental	89 ± 0.35	---	---	---	---
Streptomyces sp. strain DSD3040	*Streptomyces diastaticus*	Tubbataha Reefs	---	---	52 ± 0.07	---	---
Streptomyces sp. strain DSD1912	*Streptomyces diastaticus*	South central Visayas	---	75 ± 0.44	---	---	---
Streptomyces sp. strain DSD1631	*Streptomyces diastaticus*	Negros Occidental	75 ± 3.26	---	---	---	---
Streptomyces sp. strain DSD133	*Streptomyces ennissocaesilis*	Southeastern Iloilo	93 ± 0.54	---	---	---	---

**Isolate Code and Positive Control**	**16S rRNA Gene Sequence Match (97 to 99% identity)**	**Collection Site**	**Antibacterial Activities % Growth Inhibition ± SD** (conc. of extract = 2.5 mg/ml)				**Anticancer Activity % cytotoxic activity ± SD** (conc. of extract = 400 µg/ml)
			S. aureus ATCC BAA-44	*E. coli* ATCC 25922	*P. aeruginosa* ATCC 27853	*E. aerogenes* ATCC 13048	Ovarian Cancer (A2780)
Streptomyces sp. strain DSD164	*Streptomyces ennissocaesilis*	Southern Antique	92 ± 0.45	---	---	---	---
Streptomyces sp. strain DSD165	*Streptomyces ennissocaesilis*	Southern Antique	96 ± 0.53	---	---	---	---
Streptomyces sp. strain DSD294	*Streptomyces ennissocaesilis*	Southeastern Iloilo	94 ± 0.50	---	---	---	---
Streptomyces sp. strain DSD318	*Streptomyces ennissocaesilis*	Southeastern Iloilo	---	74 ± 2.25	89 ± 6.71	---	---
Streptomyces sp. strain DSD322	*Streptomyces ennissocaesilis*	Southern Antique	52 ± 2.03	---	---	---	72 ± 0.16
Streptomyces sp. strain DSD357	*Streptomyces ennissocaesilis*	Southern Antique	94 ± 3.18	---	---	---	---
Streptomyces sp. strain DSD360	*Streptomyces ennissocaesilis*	Southern Antique	95 ± 1.13	86 ± 0.84	---	---	---
Streptomyces sp. strain DSD800	*Streptomyces ennissocaesilis*	Western Antique	94 ± 0.16	---	---	---	---
Streptomyces sp. strain DSD998	*Streptomyces ennissocaesilis*	Western Antique	86 ± 3.64	---	---	---	51 ± 0.10
Streptomyces sp. strain DSD1986	*Streptomyces ennissocaesilis*	Western Antique	97 ± 0.16	---	---	---	---

Isolate Code and Positive Control	16S rRNA Gene Sequence Match (97 to 99% identity)	Collection Site	**Antibacterial Activities % Growth Inhibition ± SD** (conc. of extract = 2.5 mg/ml)				**Anticancer Activity % cytotoxic activity ± SD** (conc. of extract = 400 µg/ml)
			S. aureus ATCC BAA-44	*E. coli* ATCC 25922	*P. aeruginosa* ATCC 27853	*E. aerogenes* ATCC 13048	Ovarian Cancer (A2780)
Streptomyces sp. strain DSD775	*Streptomyces ennissocaesilis*	North western Antique	79 ± 5.13	---	---	---	---
Streptomyces sp. strain DSD476	*Streptomyces geysiriensis*	Southern Antique	91 ± 0.10	---	---	---	---
Streptomyces sp. strain DSD176	*Streptomyces geysiriensis*	Southern Antique	92 ± 1.40	---	---	---	---
Streptomyces sp. strain DSD342	*Streptomyces geysiriensis*	Southeastern Iloilo	56 ±10.62	---	---	---	64 ± 0.02
Streptomyces sp. strain DSD767	*Streptomyces griseorubens*	North western Antique	---	---	96 ± 1.16	---	68 ± 0.02
Streptomyces sp. strain DSD757	*Streptomyces harbinensis*	Southern Antique	95 ± 0.16	---	---	---	---
Streptomyces sp. strain DSD954	*Streptomyces harbinensis*	Northern Antique	77 ± 6.16	---	---	---	---
Streptomyces sp. strain DSD251	*Streptomyces kunmingensis*	Southern Antique	59 ± 2.71	---	---	---	61 ± 0.05
Streptomyces sp. strain DSD250	*Streptomyces kunmingensis*	Southern Antique	60 ± 4.77	59 ± 6.15	---	---	58 ± 0.02
Streptomyces sp. strain DSD1764	*Streptomyces kunmingensis*	Northern Antique	62 ± 2.83	---	---	---	73 ± 0.04

Isolate Code and Positive Control	16S rRNA Gene Sequence Match (97 to 99% identity)	Collection Site	**Antibacterial Activities % Growth Inhibition ± SD** (conc. of extract = 2.5 mg/ml)				**Anticancer Activity % cytotoxic activity ± SD** (conc. of extract = 400 µg/ml)
			S. aureus ATCC BAA-44	*E. coli* ATCC 25922	*P. aeruginosa* ATCC 27853	*E. aerogenes* ATCC 13048	Ovarian Cancer (A2780)
Streptomyces sp. strain DSD1804	*Streptomyces kunmingensis*	Northern Antique	57 ± 2.21	---	---	---	52 ± 0.10
Streptomyces sp. strain DSD3034	*Streptomyces mutabilis*	Tubbataha Reefs	64 ± 6.85	---	---	---	---
Streptomyces sp. strain DSD3030	*Streptomyces mutabilis*	Tubbataha Reefs	98 ± 0.11	---	---	---	---
Streptomyces sp. strain DSD3047	*Streptomyces mutabilis*	Tubbataha Reefs	96 ± 0.08	69 ± 8.64	---	---	---
Streptomyces sp. strain DSD2610	*Streptomyces mutabilis*	Tubbataha Reefs	93 ± 1.50	---	---	---	---
Streptomyces sp. strain DSD2613	*Streptomyces mutabilis*	Tubbataha Reefs	95 ± 2.36	---	---	---	---
Streptomyces sp. strain DSD2594	*Streptomyces mutabilis*	Tubbataha Reefs	---	---	55 ± 0.07	---	---
Streptomyces sp. strain DSD264	*Streptomyces olivaceus*	Southern Antique	---	82 ± 1.56	---	---	---
Streptomyces sp. strain DSD618	*Streptomyces olivaceus*	Southeastern Iloilo	99 ± 0.10	---	---	---	---
Streptomyces sp. strain DSD3049	*Streptomyces olivaceus*	Occidental Mindoro	---	---	52 ± 1.48	---	---
Streptomyces sp. strain DSD982	*Streptomyces parvulus*	Negros Oriental	84 ± 0.33	74 ± 1.33	---	---	62 ± 0.09

**Isolate Code and Positive Control**	**16S rRNA Gene Sequence Match (97 to 99% identity)**	**Collection Site**	**Antibacterial Activities % Growth Inhibition ± SD** (conc. of extract = 2.5 mg/ml)				**Anticancer Activity % cytotoxic activity ± SD** (conc. of extract = 400 µg/ml)
			S. aureus ATCC BAA-44	*E. coli* ATCC 25922	*P. aeruginosa* ATCC 27853	*E. aerogenes* ATCC 13048	Ovarian Cancer (A2780)
Streptomyces sp. strain DSD1639	*Streptomyces parvulus*	Negros Occidental	---	92 ± 0.01	---	---	65 ± 0.05
Streptomyces sp. strain DSD261	*Streptomyces parvulus*	Southern Antique	---	98 ± 0.00	---	---	---
Streptomyces sp. strain DSD991	*Streptomyces parvulus*	Negros Oriental	94 ± 0.54	74 ± 2.76	---	---	---
Streptomyces sp. strain DSD1719	*Streptomyces parvulus*	South central Visayas	93 ± 0.21	---	---	---	---
Streptomyces sp. strain DSD292	*Streptomyces parvulus*	Southern Antique	---	75 ± 2.11	---	---	---
Streptomyces sp. strain DSD309	*Streptomyces parvulus*	Southeastern Iloilo	72 ± 1.15	80 ± 0.53	---	---	60 ± 0.05
Streptomyces sp. strain DSD840	*Streptomyces parvulus*	Negros Oriental	94 ± 0.79	76 ± 0.01	---	---	81 ± 0.20
Streptomyces sp. strain DSD1409	*Streptomyces parvulus*	Western Antique	---	70 ± 1.20	---	---	---
Streptomyces sp. strain DSD1690	*Streptomyces parvulus*	Negros Occidental	93 ± 0.51	57 ± 8.84	---	---	---
Streptomyces sp. strain DSD985	*Streptomyces parvulus*	Negros Oriental	92 ± 1.67	91 ± 0.01	---	---	55 ± 0.10

Isolate Code and Positive Control	16S rRNA Gene Sequence Match (97 to 99% identity)	Collection Site	**Antibacterial Activities % Growth Inhibition ± SD** (conc. of extract = 2.5 mg/ml)				**Anticancer Activity % cytotoxic activity ± SD** (conc. of extract = 400 µg/ml)
			S. aureus ATCC BAA-44	*E. coli* ATCC 25922	*P. aeruginosa* ATCC 27853	*E. aerogenes* ATCC 13048	Ovarian Cancer (A2780)
Streptomyces sp. strain DSD481	*Streptomyces parvulus*	Southern Antique	92 ± 0.70	80 ± 0.93	---	---	56 ± 0.04
Streptomyces sp. strain DSD1083	*Streptomyces parvulus*	Western Antique	---	93 ± 0.19	---	---	---
Streptomyces sp. strain DSD984	*Streptomyces parvulus*	Negros Oriental	---	63 ± 0.52	---	---	58 ± 0.03
Streptomyces sp. strain DSD992	*Streptomyces parvulus*	Negros Oriental	79 ± 1.19	80 ± 1.38	---	---	71 ± 0.14
Streptomyces sp. strain DSD154	*Streptomyces parvulus*	Southern Antique	81 ± 2.39	67 ± 0.07	---	---	---
Streptomyces sp. strain DSD1636	*Streptomyces parvulus*	Negros Occidental	77 ± 2.64	68 ± 2.32	---	---	54 ± 0.14
Streptomyces sp. strain DSD1641	*Streptomyces parvulus*	Negros Occidental	81 ± 2.35	---	---	---	---
Streptomyces sp. strain DSD1643	*Streptomyces parvulus*	Negros Occidental	68 ± 2.43	85 ± 1.26	---	---	---
Streptomyces sp. strain DSD995	*Streptomyces parvulus*	Negros Oriental	61 ± 0.11	59 ± 0.12	---	---	57 ± 0.04
Streptomyces sp. strain DSD1642	*Streptomyces parvulus*	Negros Occidental	76 ± 0.07	---	---	---	59 ± 0.04
Streptomyces sp. strain DSD3100	*Streptomyces parvulus*	Occidental Mindoro	95 ± 1.47	---	---	---	---

Isolate Code and Positive Control	16S rRNA Gene Sequence Match (97 to 99% identity)	Collection Site	Antibacterial Activities % Growth Inhibition ± SD (conc. of extract = 2.5 mg/ml)				Anticancer Activity % cytotoxic activity ± SD (conc. of extract = 400 µg/ml)
			S. aureus ATCC BAA-44	*E. coli* ATCC 25922	*P. aeruginosa* ATCC 27853	*E. aerogenes* ATCC 13048	Ovarian Cancer (A2780)
Streptomyces sp. strain DSD1637	*Streptomyces parvulus*	Negros Occidental	96 ± 0.73	94 ± 0.63	64 ± 0.87	---	---
Streptomyces sp. strain DSD1629	*Streptomyces parvulus*	Negros Occidental	92 ± 0.97	94 ± 0.36	---	---	55 ± 0.07
Streptomyces sp. strain DSD988	*Streptomyces parvulus*	Negros Oriental	61 ± 0.18	---	---	---	51 ± 0.06
Streptomyces sp. strain DSD986	*Streptomyces parvulus*	Negros Oriental	---	55 ± 12.83	---	---	58 ± 0.03
Streptomyces sp. strain DSD993	*Streptomyces parvulus*	Negros Oriental	62 ± 0.09	---	---	---	57 ± 0.02
Streptomyces sp. strain DSD1072	*Streptomyces parvulus*	Western Antique	---	86 ± 3.06	---	---	---
Streptomyces sp. strain DSD1969	*Streptomyces parvulus*	South central Visayas	67 ± 1.49	51 ± 0.08	---	---	---
Streptomyces sp. strain DSD1692	*Streptomyces parvulus*	Negros Occidental	50 ± 0.50	---	---	---	67 ± 0.04
Streptomyces sp. strain DSD1632	*Streptomyces parvulus*	Negros Occidental	---	70 ± 3.16	---	---	59 ± 0.09
Streptomyces sp. strain DSD1628	*Streptomyces parvulus*	Negros Occidental	85 ± 0.07	85 ± 1.50	---	---	64 ± 0.05

Isolate Code and Positive Control	16S rRNA Gene Sequence Match (97 to 99% identity)	Collection Site	**Antibacterial Activities % Growth Inhibition ± SD** (conc. of extract = 2.5 mg/ml)				**Anticancer Activity % cytotoxic activity ± SD** (conc. of extract = 400 µg/ml)
			S. aureus ATCC BAA-44	*E. coli* ATCC 25922	*P. aeruginosa* ATCC 27853	*E. aerogenes* ATCC 13048	Ovarian Cancer (A2780)
Streptomyces sp. strain DSD2596	*Streptomyces parvulus*	Occidental Mindoro	77 ± 4.14	---	---	---	57 ± 0.05
Streptomyces sp. strain DSD2603	*Streptomyces pseudogriseolus*	Tubbataha Reefs	95 ± 0.00	---	---	---	---
Streptomyces sp. strain DSD120	*Streptomyces rochei*	Southeastern Iloilo	90 ± 1.24	---	---	---	---
Streptomyces sp. strain DSD243	*Streptomyces rochei*	Southern Antique	86 ± 2.34	---	---	---	---
Streptomyces sp. strain DSD284	*Streptomyces rochei*	Southern Antique	96 ± 0.08	---	---	---	57 ± 0.04
Streptomyces sp. strain DSD323	*Streptomyces rochei*	Southern Antique	89 ± 0.32	---	---	---	---
Streptomyces sp. strain DSD501	*Streptomyces rochei*	Northern Antique	94 ± 1.96	---	---	---	59 ± 0.06
Streptomyces sp. strain DSD839	*Streptomyces rochei*	Negros Oriental	---	50 ± 0.01	---	---	---
Streptomyces sp. strain DSD1328	*Streptomyces rochei*	Central Visayas	94 ± 0.31	---	---	---	52 ± 0.06
Streptomyces sp. strain DSD1342	*Streptomyces rochei*	Negros Occidental	89 ± 0.39	---	---	---	---
Streptomyces sp. strain DSD1381	*Streptomyces rochei*	Negros Occidental	96 ± 0.64	---	---	---	55 ± 0.09

**Isolate Code and Positive Control**	**16S rRNA Gene Sequence Match (97 to 99% identity)**	**Collection Site**	**Antibacterial Activities % Growth Inhibition ± SD** (conc. of extract = 2.5 mg/ml)				**Anticancer Activity % cytotoxic activity ± SD** (conc. of extract = 400 µg/ml)
			S. aureus ATCC BAA-44	*E. coli* ATCC 25922	*P. aeruginosa* ATCC 27853	*E. aerogenes* ATCC 13048	Ovarian Cancer (A2780)
Streptomyces sp. strain DSD1555	*Streptomyces rochei*	Negros Oriental	96 ± 1.65	66 ± 5.97	---	---	---
Streptomyces sp. strain DSD2625	*Streptomyces rochei*	Occidental Mindoro	67 ± 4.18	---	---	---	---
Streptomyces sp. strain DSD3018	*Streptomyces sedi*	Occidental Mindoro	57 ± 2.74	---	---	---	71 ± 0.07
Streptomyces sp. strain DSD3011	*Streptomyces sedi*	Occidental Mindoro	86 ± 1.82	90 ± 10.9	---	---	---
Streptomyces sp. strain DSD2987	*Streptomyces sedi*	Occidental Mindoro	77 ± 2.88	53 ± 3.52	---	---	---
Streptomyces sp. strain DSD977	*Streptomyces xiamenensis*	Western Antique	83 ± 0.94	---	---	---	61 ± 0.07
Streptomyces sp. strain DSD742	*Streptomyces* sp. nov.	Western Antique	---	100 ± 0.19	---	---	68 ± 0.02
Streptomyces sp. strain DSD3025	*Streptomyces* sp. nov.	Tubbataha Reefs	97 ± 0.00	---	---	---	---
Streptomyces sp. strain DSD1006	*Streptomyces* sp. nov.	Northern Antique	92 ± 0.61	---	---	---	---
Tetracycline (0.25 mg/mL)			98 ± 0.07	98 ± 0.07	99 ± 0.04	99 ± 0.02	

---, not active

Supplementary Table S5. NCBI GenBank Accession Numbers of 16S rRNA and *rpoB* Gene Sequences

Streptomyces Strain Code	Collection Site	16s rRNA			rpoB		
		Accession number	Date Released	Accession URL	Accession Number	Date Released	Accession URL
**DSD1715**	Western Antique	MW217110	03/31/2021	https://www.ncbi.nlm.nih.gov/nuccore/MW217110.1	MW820643	07/26/2021	https://www.ncbi.nlm.nih.gov/nuccore/MW820643.1
**DSD2595**	Tubbataha Reefs	MW217135	03/31/2021	https://www.ncbi.nlm.nih.gov/nuccore/MW217135.1	MW855583	07/26/2021	https://www.ncbi.nlm.nih.gov/nuccore/MW855583.1
**DSD274**	Southeastern Iloilo	MW217097	03/31/2021	https://www.ncbi.nlm.nih.gov/nuccore/MW217097.1	MW855584	07/26/2021	https://www.ncbi.nlm.nih.gov/nuccore/MW855584.1
**DSD331**	Southeastern Iloilo	MW217113	03/31/2021	https://www.ncbi.nlm.nih.gov/nuccore/MW217113.1	MW831619	07/26/2021	https://www.ncbi.nlm.nih.gov/nuccore/MW831619.1
**DSD299**	Southeastern Iloilo	MW217181	03/31/2021	https://www.ncbi.nlm.nih.gov/nuccore/MW217181.1	MW776874	07/26/2021	https://www.ncbi.nlm.nih.gov/nuccore/MW776874.1
**DSD1631**	Negros Occidental	MW217167	03/31/2021	https://www.ncbi.nlm.nih.gov/nuccore/MW217167.1	MW776875	07/26/2021	https://www.ncbi.nlm.nih.gov/nuccore/MW776875.1
**DSD1886**	Tubbataha Reefs	MW217079	03/31/2021	https://www.ncbi.nlm.nih.gov/nuccore/1928656540	MW776876	07/26/2021	https://www.ncbi.nlm.nih.gov/nuccore/MW776876.1
**DSD1912**	South central Visayas	MW217108	03/31/2021	https://www.ncbi.nlm.nih.gov/nuccore/MW217108.1	MW855585	07/26/2021	https://www.ncbi.nlm.nih.gov/nuccore/MW855585.1
**DSD3040**	Negros Occidental	MW217151	03/31/2021	https://www.ncbi.nlm.nih.gov/nuccore/MW217151.1	MW883337	07/26/2021	https://www.ncbi.nlm.nih.gov/nuccore/MW883337.1
**DSD133**	Southeastern Iloilo	MW217123	03/31/2021	https://www.ncbi.nlm.nih.gov/nuccore/MW217123.1	MW820618	07/28/2021	https://www.ncbi.nlm.nih.gov/nuccore/MW820618.1

Streptomyces Strain Code	Collection Site	16s rRNA			rpoB		
		Accession number	Date Released	Accession URL	Accession Number	Date Released	Accession URL
**DSD164**	Southern Antique	MW217124	03/31/2021	https://www.ncbi.nlm.nih.gov/nuccore/MW217124.1	MW820619	07/28/2021	https://www.ncbi.nlm.nih.gov/nuccore/MW820619.1
**DSD165**	Southern Antique	MW217125	03/31/2021	https://www.ncbi.nlm.nih.gov/nuccore/MW217125.1	MW725554	07/26/2021	https://www.ncbi.nlm.nih.gov/nuccore/MW725554.1
**DSD294**	Southeastern Iloilo	MW217127	03/31/2021	https://www.ncbi.nlm.nih.gov/nuccore/MW217127.1	MW979248	07/26/2021	https://www.ncbi.nlm.nih.gov/nuccore/MW979248.1
**DSD318**	Southeastern Iloilo	MW217112	03/31/2021	https://www.ncbi.nlm.nih.gov/nuccore/MW217112.1	MW922317	07/26/2021	https://www.ncbi.nlm.nih.gov/nuccore/MW922317.1
**DSD322**	Southern Antique	MW217140	03/31/2021	https://www.ncbi.nlm.nih.gov/nuccore/MW217140.1	MW725555	07/26/2021	https://www.ncbi.nlm.nih.gov/nuccore/MW725555.1
**DSD357**	Southern Antique	MW217128	03/31/2021	https://www.ncbi.nlm.nih.gov/nuccore/MW217128.1	MW725556	07/26/2021	https://www.ncbi.nlm.nih.gov/nuccore/MW725556.1
**DSD360**	Southern Antique	MW217134	03/31/2021	https://www.ncbi.nlm.nih.gov/nuccore/MW217134.1	MW820621	07/28/2021	https://www.ncbi.nlm.nih.gov/nuccore/MW820621.1
**DSD775**	Western Antique	MW217138	03/31/2021	https://www.ncbi.nlm.nih.gov/nuccore/1928656595	MW831606	07/26/2021	https://www.ncbi.nlm.nih.gov/nuccore/MW831606.1
**DSD800**	Western Antique	MW217114	03/31/2021	https://www.ncbi.nlm.nih.gov/nuccore/MW217114.1	MW776842	07/26/2021	https://www.ncbi.nlm.nih.gov/nuccore/MW776842.1
**DSD998**	Western Antique	MW217115	03/31/2021	https://www.ncbi.nlm.nih.gov/nuccore/MW217115.1	MW776843	07/26/2021	https://www.ncbi.nlm.nih.gov/nuccore/MW776843.1

Streptomyces Strain Code	Collection Site	16s rRNA			rpoB		
		**Accession number**	**Date Released**	**Accession URL**	**Accession Number**	**Date Released**	**Accession URL**
**DSD1986**	North western Antique	MW217101	03/31/2021	https://www.ncbi.nlm.nih.gov/nuccore/MW217101.1	MW725557	07/26/2021	https://www.ncbi.nlm.nih.gov/nuccore/MW725557.1
**DSD176**	Southern Antique	MW217126	03/31/2021	https://www.ncbi.nlm.nih.gov/nuccore/MW217126.1	MW693208	07/26/2021	https://www.ncbi.nlm.nih.gov/nuccore/MW693208.1
**DSD342**	Southern Antique	MW217081	03/31/2021	https://www.ncbi.nlm.nih.gov/nuccore/MW217081.1	MW855586	07/26/2021	https://www.ncbi.nlm.nih.gov/nuccore/MW855586.1
**DSD476**	Southeastern Iloilo	MW217161	03/31/2021	https://www.ncbi.nlm.nih.gov/nuccore/MW217161.1	MW855587	07/26/2021	https://www.ncbi.nlm.nih.gov/nuccore/MW855587.1
**DSD767**	North western Antique	MW217085	03/31/2021	https://www.ncbi.nlm.nih.gov/nuccore/MW217085.1	MW820645	07/26/2021	https://www.ncbi.nlm.nih.gov/nuccore/MW820645.1
**DSD757**	Southern Antique	MW217068	03/31/2021	https://www.ncbi.nlm.nih.gov/nuccore/MW217068.1	MW855588	07/26/2021	https://www.ncbi.nlm.nih.gov/nuccore/MW855588.1
**DSD954**	Northern Antique	MW217089	03/31/2021	https://www.ncbi.nlm.nih.gov/nuccore/MW217089.1	MW883338	07/26/2021	https://www.ncbi.nlm.nih.gov/nuccore/MW883338.1
**DSD250**	Southern Antique	MW217165	03/31/2021	https://www.ncbi.nlm.nih.gov/nuccore/MW217098.1	MW831618	07/26/2021	https://www.ncbi.nlm.nih.gov/nuccore/MW831618.1
**DSD251**	Southern Antique	MW217098	03/31/2021	https://www.ncbi.nlm.nih.gov/nuccore/MW217165.1	MW776871	07/26/2021	https://www.ncbi.nlm.nih.gov/nuccore/MW776871.1
**DSD1764**	Northern Antique	MW217139	03/31/2021	https://www.ncbi.nlm.nih.gov/nuccore/MW217139.1	MW776872	07/26/2021	https://www.ncbi.nlm.nih.gov/nuccore/MW776872.1

Streptomyces Strain Code	Collection Site	16s rRNA			rpoB		
		Accession number	Date Released	Accession URL	Accession Number	Date Released	Accession URL
**DSD1804**	Northern Antique	MW217083	03/31/2021	https://www.ncbi.nlm.nih.gov/nuccore/MW217083.1	MW776873	07/26/2021	https://www.ncbi.nlm.nih.gov/nuccore/MW776873.1
**DSD2594**	Tubbataha Reefs	MW217102	03/31/2021	https://www.ncbi.nlm.nih.gov/nuccore/MW217102.1	MW831617	07/26/2021	https://www.ncbi.nlm.nih.gov/nuccore/MW831617.1
**DSD2610**	Tubbataha Reefs	MW217073	03/31/2021	https://www.ncbi.nlm.nih.gov/nuccore/MW217073.1	MW820646	07/26/2021	https://www.ncbi.nlm.nih.gov/nuccore/MW820646.1
**DSD2613**	Tubbataha Reefs	MW217194	03/31/2021	https://www.ncbi.nlm.nih.gov/nuccore/MW217194.1	MW820644	07/26/2021	https://www.ncbi.nlm.nih.gov/nuccore/MW820644.1
**DSD3030**	Tubbataha Reefs	MW217120	03/31/2021	https://www.ncbi.nlm.nih.gov/nuccore/MW217120.1	MW776870	07/26/2021	https://www.ncbi.nlm.nih.gov/nuccore/MW776870.1
**DSD3034**	Tubbataha Reefs	MW217121	03/31/2021	https://www.ncbi.nlm.nih.gov/nuccore/MW217121.1	MW820642	07/26/2021	https://www.ncbi.nlm.nih.gov/nuccore/MW820642.1
**DSD3047**	Tubbataha Reefs	MW217195	03/31/2021	https://www.ncbi.nlm.nih.gov/nuccore/MW217195.1	MW820620	07/28/2021	https://www.ncbi.nlm.nih.gov/nuccore/MW820620.1
**DSD264**	Southern Antique	MW217164	03/31/2021	https://www.ncbi.nlm.nih.gov/nuccore/MW217164.1	MW855589	07/26/2021	https://www.ncbi.nlm.nih.gov/nuccore/MW855589.1
**DSD618**	Southeastern Iloilo	MW217084	03/31/2021	https://www.ncbi.nlm.nih.gov/nuccore/MW217084.1	MW725549	07/26/2021	https://www.ncbi.nlm.nih.gov/nuccore/MW725549.1
**DSD3049**	Occidental Mindoro	MW217071	03/31/2021	https://www.ncbi.nlm.nih.gov/nuccore/MW217071.1	MW820641	07/26/2021	https://www.ncbi.nlm.nih.gov/nuccore/MW820641.1

Streptomyces Strain Code	Collection Site	16s rRNA			rpoB		
		Accession number	Date Released	Accession URL	Accession Number	Date Released	Accession URL
**DSD154**	Negros Oriental	MW217166	03/31/2021	https://www.ncbi.nlm.nih.gov/nuccore/MW217166.1	MW831608	07/26/2021	https://www.ncbi.nlm.nih.gov/nuccore/MW831608.1
**DSD261**	Negros Occidental	MW217147	03/31/2021	https://www.ncbi.nlm.nih.gov/nuccore/MW217147.1	MW767611	07/26/2021	https://www.ncbi.nlm.nih.gov/nuccore/MW767611.1
**DSD292**	Southern Antique	MW217162	03/31/2021	https://www.ncbi.nlm.nih.gov/nuccore/MW217162.1	MW753172	07/28/2021	https://www.ncbi.nlm.nih.gov/nuccore/MW753172.1
**DSD309**	Negros Oriental	MW217086	03/31/2021	https://www.ncbi.nlm.nih.gov/nuccore/MW217086.1	MW767616	07/26/2021	https://www.ncbi.nlm.nih.gov/nuccore/MW767616.1
**DSD481**	South central Visayas	MW217129	03/31/2021	https://www.ncbi.nlm.nih.gov/nuccore/MW217129.1	MW820612	07/28/2021	https://www.ncbi.nlm.nih.gov/nuccore/MW820612.1
**DSD840**	Southern Antique	MW217160	03/31/2021	https://www.ncbi.nlm.nih.gov/nuccore/MW217160.1	MW767615	07/26/2021	https://www.ncbi.nlm.nih.gov/nuccore/MW767615.1
**DSD982**	Southeastern Iloilo	MW217122	03/31/2021	https://www.ncbi.nlm.nih.gov/nuccore/MW217122.1	MW725558	07/26/2021	https://www.ncbi.nlm.nih.gov/nuccore/MW725558.1
**DSD984**	Negros Oriental	MW217111	03/31/2021	https://www.ncbi.nlm.nih.gov/nuccore/MW217111.1	MW767614	07/26/2021	https://www.ncbi.nlm.nih.gov/nuccore/MW767614.1
**DSD985**	Western Antique	MW217130	03/31/2021	https://www.ncbi.nlm.nih.gov/nuccore/MW217130.1	MW753175	07/28/2021	https://www.ncbi.nlm.nih.gov/nuccore/MW753175.1
**DSD986**	Negros Occidental	MW217182	03/31/2021	https://www.ncbi.nlm.nih.gov/nuccore/MW217182.1	MW767613	07/26/2021	https://www.ncbi.nlm.nih.gov/nuccore/MW767613.1

Streptomyces Strain Code	Collection Site	16s rRNA			rpoB		
		Accession number	Date Released	Accession URL	Accession Number	Date Released	Accession URL
**DSD988**	Negros Oriental	MW217159	03/31/2021	https://www.ncbi.nlm.nih.gov/nuccore/MW217159.1	MW855580	07/26/2021	https://www.ncbi.nlm.nih.gov/nuccore/MW855580.1
**DSD991**	Southern Antique	MW217145	03/31/2021	https://www.ncbi.nlm.nih.gov/nuccore/MW217145.1	MW753170	07/28/2021	https://www.ncbi.nlm.nih.gov/nuccore/MW753170.1
**DSD992**	Western Antique	MW217141	03/31/2021	https://www.ncbi.nlm.nih.gov/nuccore/MW217141.1	MW753176	07/28/2021	https://www.ncbi.nlm.nih.gov/nuccore/MW753176.1
**DSD993**	Negros Oriental	MW217183	03/31/2021	https://www.ncbi.nlm.nih.gov/nuccore/MW217183.1	MW831614	07/26/2021	https://www.ncbi.nlm.nih.gov/nuccore/MW831614.1
**DSD995**	Negros Oriental	MW217148	03/31/2021	https://www.ncbi.nlm.nih.gov/nuccore/MW217148.1	MW831612	07/26/2021	https://www.ncbi.nlm.nih.gov/nuccore/?term=dsd995
**DSD1072**	Southern Antique	MW217075	03/31/2021	https://www.ncbi.nlm.nih.gov/nuccore/MW217075.1	MW767612	07/26/2021	https://www.ncbi.nlm.nih.gov/nuccore/MW767612.1
**DSD1083**	Negros Occidental	MW217090	03/31/2021	https://www.ncbi.nlm.nih.gov/nuccore/MW217090.1	MW820639	07/26/2021	https://www.ncbi.nlm.nih.gov/nuccore/MW820639.1
**DSD1409**	Negros Occidental	MW217158	03/31/2021	https://www.ncbi.nlm.nih.gov/nuccore/MW217158.1	MW831607	07/26/2021	https://www.ncbi.nlm.nih.gov/nuccore/MW831607.1
**DSD1628**	Negros Occidental	MW217189	03/31/2021	https://www.ncbi.nlm.nih.gov/nuccore/MW217189.1	MW855581	07/26/2021	https://www.ncbi.nlm.nih.gov/nuccore/MW855581.1
**DSD1629**	Negros Oriental	MW217107	03/31/2021	https://www.ncbi.nlm.nih.gov/nuccore/MW217107.1	MW753178	07/28/2021	https://www.ncbi.nlm.nih.gov/nuccore/MW753178.1

Streptomyces Strain Code	Collection Site	16s rRNA			rpoB		
		Accession number	Date Released	Accession URL	Accession Number	Date Released	Accession URL
**DSD1632**	Negros Occidental	MW217190	03/31/2021	https://www.ncbi.nlm.nih.gov/nuccore/MW217190.1	MW855582	07/26/2021	https://www.ncbi.nlm.nih.gov/nuccore/MW855582.1
**DSD1636**	Occidental Mindoro	MW217092	03/31/2021	https://www.ncbi.nlm.nih.gov/nuccore/MW217092.1	MW831609	07/26/2021	https://www.ncbi.nlm.nih.gov/nuccore/MW831609.1
**DSD1637**	Negros Occidental	MW217105	03/31/2021	https://www.ncbi.nlm.nih.gov/nuccore/MW217105.1	MW753177	07/28/2021	https://www.ncbi.nlm.nih.gov/nuccore/MW753177.1
**DSD1639**	Negros Occidental	MW217177	03/31/2021	https://www.ncbi.nlm.nih.gov/nuccore/MW217177.1	MW776844	07/26/2021	https://www.ncbi.nlm.nih.gov/nuccore/MW776844.1
**DSD1641**	Negros Oriental	MW217104	03/31/2021	https://www.ncbi.nlm.nih.gov/nuccore/MW217104.1	MW831610	07/26/2021	https://www.ncbi.nlm.nih.gov/nuccore/MW831610.1
**DSD1642**	Negros Oriental	MW217106	03/31/2021	https://www.ncbi.nlm.nih.gov/nuccore/MW217106.1	MW820640	07/26/2021	https://www.ncbi.nlm.nih.gov/nuccore/MW820640.1
**DSD1643**	Negros Oriental	MW217176	03/31/2021	https://www.ncbi.nlm.nih.gov/nuccore/MW217176.1	MW831611	07/26/2021	https://www.ncbi.nlm.nih.gov/nuccore/MW831611.1
**DSD1690**	Western Antique	MW217146	03/31/2021	https://www.ncbi.nlm.nih.gov/nuccore/MW217146.1	MW753174	07/28/2021	https://www.ncbi.nlm.nih.gov/nuccore/MW753174.1
**DSD1692**	South central Visayas	MW217191	03/31/2021	https://www.ncbi.nlm.nih.gov/nuccore/MW217191.1	MW831616	07/26/2021	https://www.ncbi.nlm.nih.gov/nuccore/MW831616.1
**DSD1719**	Negros Occidental	MW217109	03/31/2021	https://www.ncbi.nlm.nih.gov/nuccore/MW217109.1	MW753171	07/28/2021	https://www.ncbi.nlm.nih.gov/nuccore/MW753171.1

Streptomyces Strain Code	Collection Site	16s rRNA			rpoB		
		Accession number	Date Released	Accession URL	Accession Number	Date Released	Accession URL
**DSD1969**	Negros Occidental	MW217133	03/31/2021	https://www.ncbi.nlm.nih.gov/nuccore/MW217133.1	MW831615	07/26/2021	https://www.ncbi.nlm.nih.gov/nuccore/MW831615.1
**DSD2596**	Negros Occidental	MW217198	03/31/2021	https://www.ncbi.nlm.nih.gov/nuccore/MW217198.1	MW776869	07/26/2021	https://www.ncbi.nlm.nih.gov/nuccore/MW776869.1
**DSD3100**	Occidental Mindoro	MW217072	03/31/2021	https://www.ncbi.nlm.nih.gov/nuccore/MW217072.1	MW831613	07/26/2021	https://www.ncbi.nlm.nih.gov/nuccore/MW831613.1
**DSD2603**	Tubbataha Reefs	MW217103	03/31/2021	https://www.ncbi.nlm.nih.gov/nuccore/MW217103.1	MW855590	07/26/2021	https://www.ncbi.nlm.nih.gov/nuccore/MW855590.1
**DSD120**	Southeastern Iloilo	MW217172	03/31/2021	https://www.ncbi.nlm.nih.gov/nuccore/MW217172.1	MW725550	07/26/2021	https://www.ncbi.nlm.nih.gov/nuccore/MW725550.1
**DSD243**	Southern Antique	MW217136	03/31/2021	https://www.ncbi.nlm.nih.gov/nuccore/MW217136.1	MW820613	07/28/2021	https://www.ncbi.nlm.nih.gov/nuccore/MW820613.1
**DSD284**	Southern Antique	MW217163	03/31/2021	https://www.ncbi.nlm.nih.gov/nuccore/MW217163.1	MW725551	07/26/2021	https://www.ncbi.nlm.nih.gov/nuccore/MW725551.1
**DSD323**	Southern Antique	MW217078	03/31/2021	https://www.ncbi.nlm.nih.gov/nuccore/MW217078.1	MW693209	07/26/2021	https://www.ncbi.nlm.nih.gov/nuccore/MW693209.1
**DSD501**	Northern Antique	MW217088	03/31/2021	https://www.ncbi.nlm.nih.gov/nuccore/MW217088.1	MW820614	07/28/2021	https://www.ncbi.nlm.nih.gov/nuccore/MW820614.1
**DSD839**	Negros Oriental	MW217173	03/31/2021	https://www.ncbi.nlm.nih.gov/nuccore/MW217173.1	MW725552	07/26/2021	https://www.ncbi.nlm.nih.gov/nuccore/MW725552.1

Streptomyces Strain Code	Collection Site	16s rRNA			rpoB		
		Accession number	Date Released	Accession URL	Accession Number	Date Released	Accession URL
**DSD1328**	Central Visayas	MW217074	03/31/2021	https://www.ncbi.nlm.nih.gov/nuccore/MW217074.1	MW883334	07/26/2021	https://www.ncbi.nlm.nih.gov/nuccore/MW883334.1
**DSD1342**	Negros Occidental	MW217174	03/31/2021	https://www.ncbi.nlm.nih.gov/nuccore/MW217174.1	MW725553	07/26/2021	https://www.ncbi.nlm.nih.gov/nuccore/MW725553.1
**DSD1381**	Negros Occidental	MW217175	03/31/2021	https://www.ncbi.nlm.nih.gov/nuccore/MW217175.1	MW820615	07/28/2021	https://www.ncbi.nlm.nih.gov/nuccore/MW820615.1
**DSD1555**	Negros Oriental	MW217091	03/31/2021	https://www.ncbi.nlm.nih.gov/nuccore/MW217091.1	MW820617	07/28/2021	https://www.ncbi.nlm.nih.gov/nuccore/MW820617.1
**DSD2625**	Occidental Mindoro	MW217080	03/31/2021	https://www.ncbi.nlm.nih.gov/nuccore/MW217080.1	MW820616	07/28/2021	https://www.ncbi.nlm.nih.gov/nuccore/MW820616.1
**DSD2987**	Occidental Mindoro	MW217096	03/31/2021	https://www.ncbi.nlm.nih.gov/nuccore/MW217096.1	MW883335	07/26/2021	https://www.ncbi.nlm.nih.gov/nuccore/MW883335.1
**DSD3011**	Occidental Mindoro	MW217087	03/31/2021	https://www.ncbi.nlm.nih.gov/nuccore/MW217087.1	MW883336	07/26/2021	https://www.ncbi.nlm.nih.gov/nuccore/MW883336.1
**DSD3018**	Occidental Mindoro	MW217095	03/31/2021	https://www.ncbi.nlm.nih.gov/nuccore/1928656556	MW753179	08/01/2021	https://www.ncbi.nlm.nih.gov/nuccore/MW753179
**DSD977**	Western Antique	MW217188	03/31/2021	https://www.ncbi.nlm.nih.gov/nuccore/MW217188.1	MW693207	07/26/2021	https://www.ncbi.nlm.nih.gov/nuccore/MW693207.1
**DSD742**	Western Antique	MW048781.1	03/31/2021	https://www.ncbi.nlm.nih.gov/nuccore/MW693206.1	MW693206	07/26/2021	https://www.ncbi.nlm.nih.gov/nuccore/MW693206.1

Streptomyces Strain Code	Collection Site	16s rRNA			rpoB		
		Accession number	Date Released	Accession URL	Accession Number	Date Released	Accession URL
**DSD1006**	Tubbataha Reefs	MW116661.1	03/31/2021	https://www.ncbi.nlm.nih.gov/nuccore/MW602150.1	MW602150	07/26/2021	https://www.ncbi.nlm.nih.gov/nuccore/MW116729.1
**DSD3025**	Northern Antique	MW116729.1	03/31/2021	https://www.ncbi.nlm.nih.gov/nuccore/MW116661.1	MW246168	07/26/2021	https://www.ncbi.nlm.nih.gov/nuccore/MW246168.1

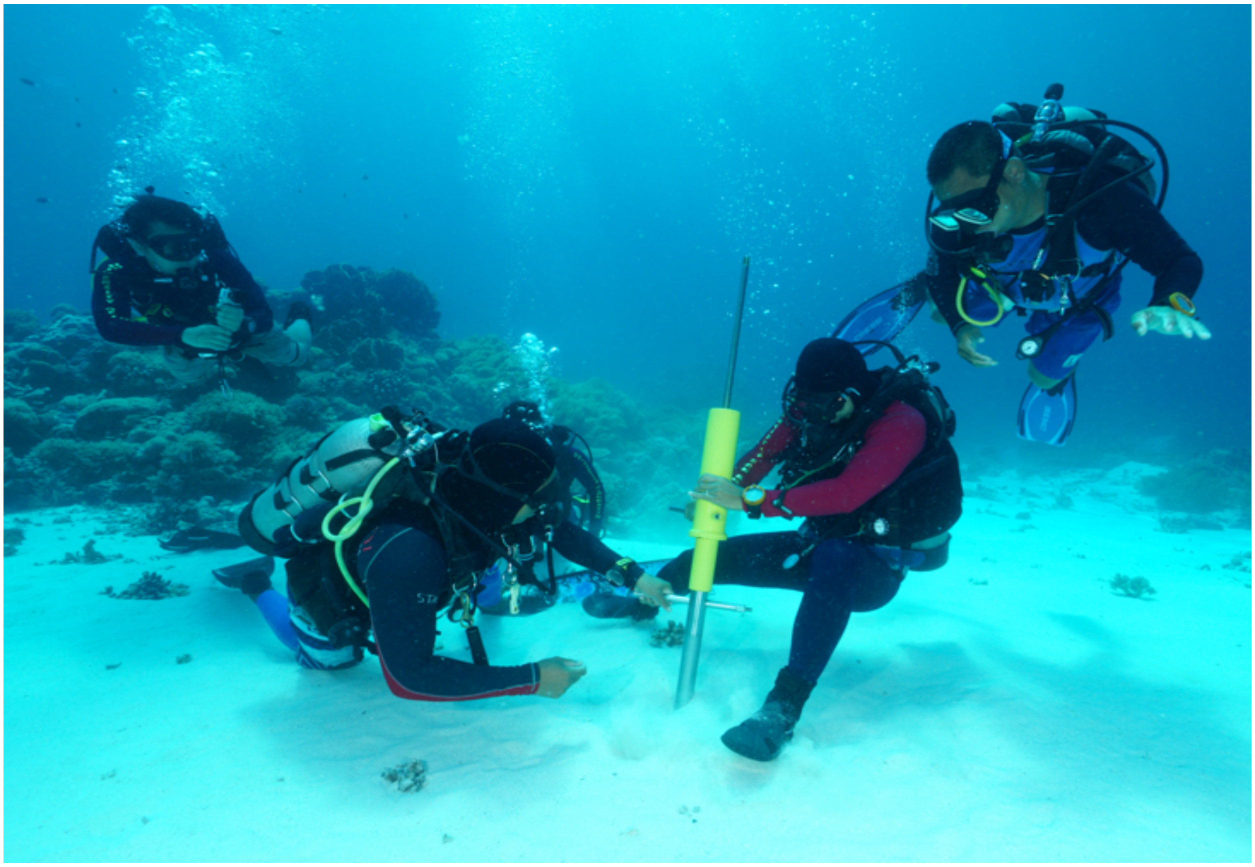

Supplementary Figure S1. Sediment Core Sampling Using a Fabricated 1-Meter Sediment Core Sampler. Marine sediments were collected by manually inserting the piston to the seabed for the core barrel to penetrate through hammering strokes.

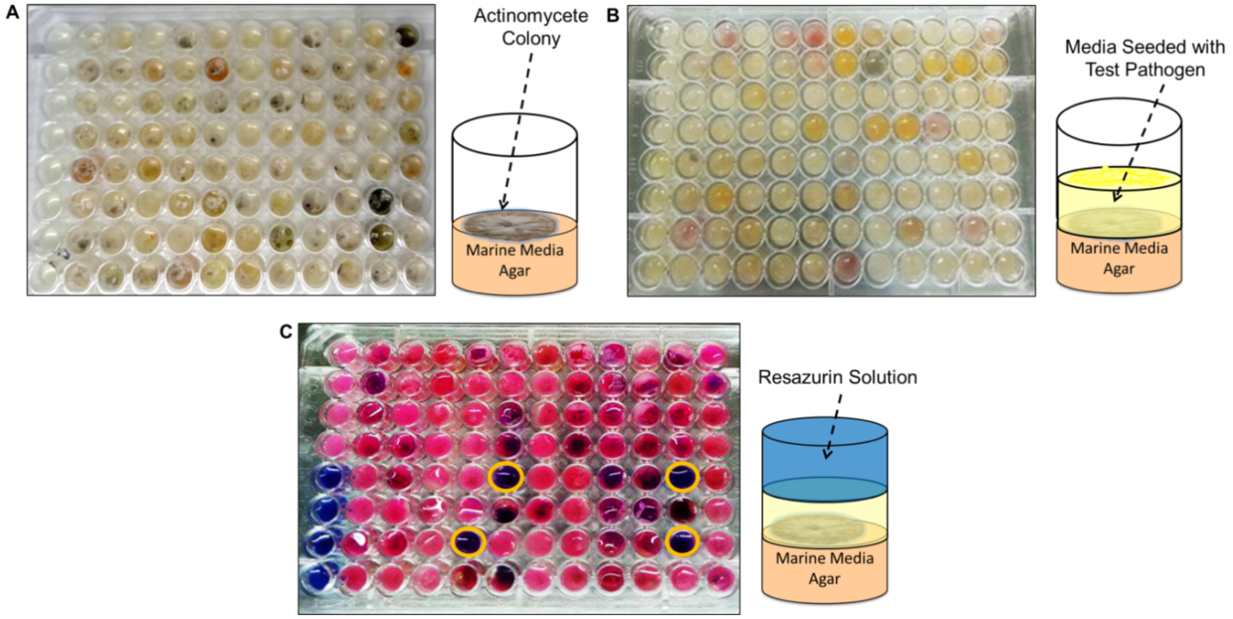

Supplementary Figure S2. Initial Antibacterial Testing of Live Cells of Actinomycete Strains Overlaid with Bacterial Suspension of Test Pathogens. **A)** 96-well plate containing marine medium agar inoculated with actinomycete. **B)** Bacterial suspension of the test pathogen was overlaid on top of the marine medium agar with actinomycete after 14 days of incubation. **C)** After 24 h of incubation at 37°C, 20 µL resazurin dye (0.2 mg/mL in PBS) was added to each well. After 10-20 min of incubation at room temperature, retained blue color of resazurin as shown by the encircled wells indicated an antibacterial activity, while the pink color signified reduction of resazurin to resorufin, indicating inactivity against test pathogen.

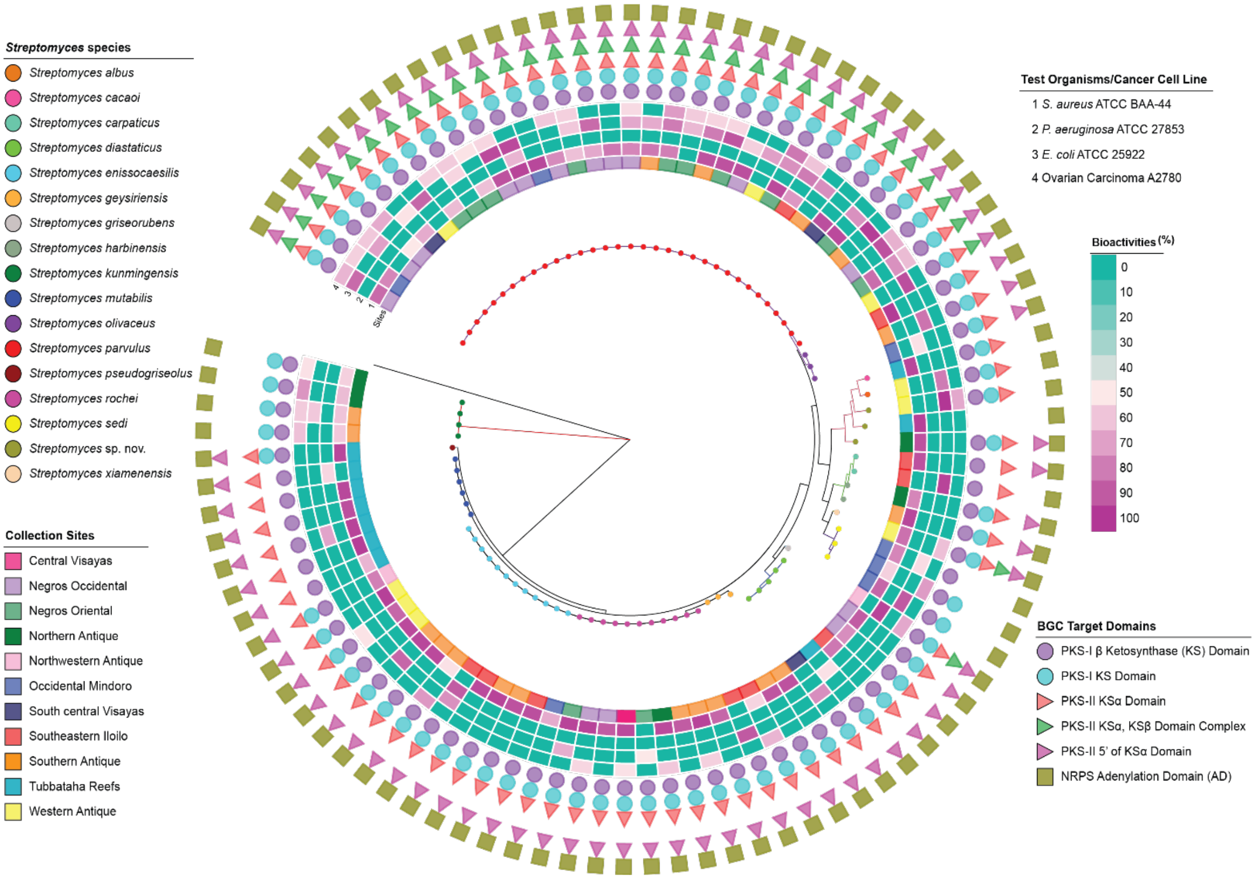

Supplementary Figure S3. Representative Distribution of Active *Streptomyces* in Philippine Sampling Sites Based on the 16S rRNA Gene Sequences, and their Detected BGCs in Real Time-PCR. The phylogenetic tree of *Streptomyces* was constructed using 16S rRNA gene sequences. Different species were represented by colored circles in the tree. Clusters indicated by colored nodes in the tree were selected based on ≥90% bootstrap replicates. The collection sites of strains were annotated by the color strips adjacent to the tree. Detected BGCs using PKS and NRPS primer sets were represented by the colored squares, circles, and triangles. Heat map in the outermost portion of tree displayed the antibacterial and anticancer activities of strains against the four test pathogens and ovarian cancer cell line (A2780). Color gradient ranging from purple (90-100% activity) to blue-green (no activity) corresponds to the bioactivities of strains. The phylogenetic tree was generated with maximum-likelihood algorithms using mega 7.0 (1000 bootstrap replications) and the substitution model Tamura–Nei method. The tree is drawn to scale, with branch lengths in the same units as those of the evolutionary distances used to infer the phylogenetic tree. The tree was visualized and annotated using interactive tree of life (iTOL v4 5.6.3 (Biobyte solutions, Heidelberg, Germany).

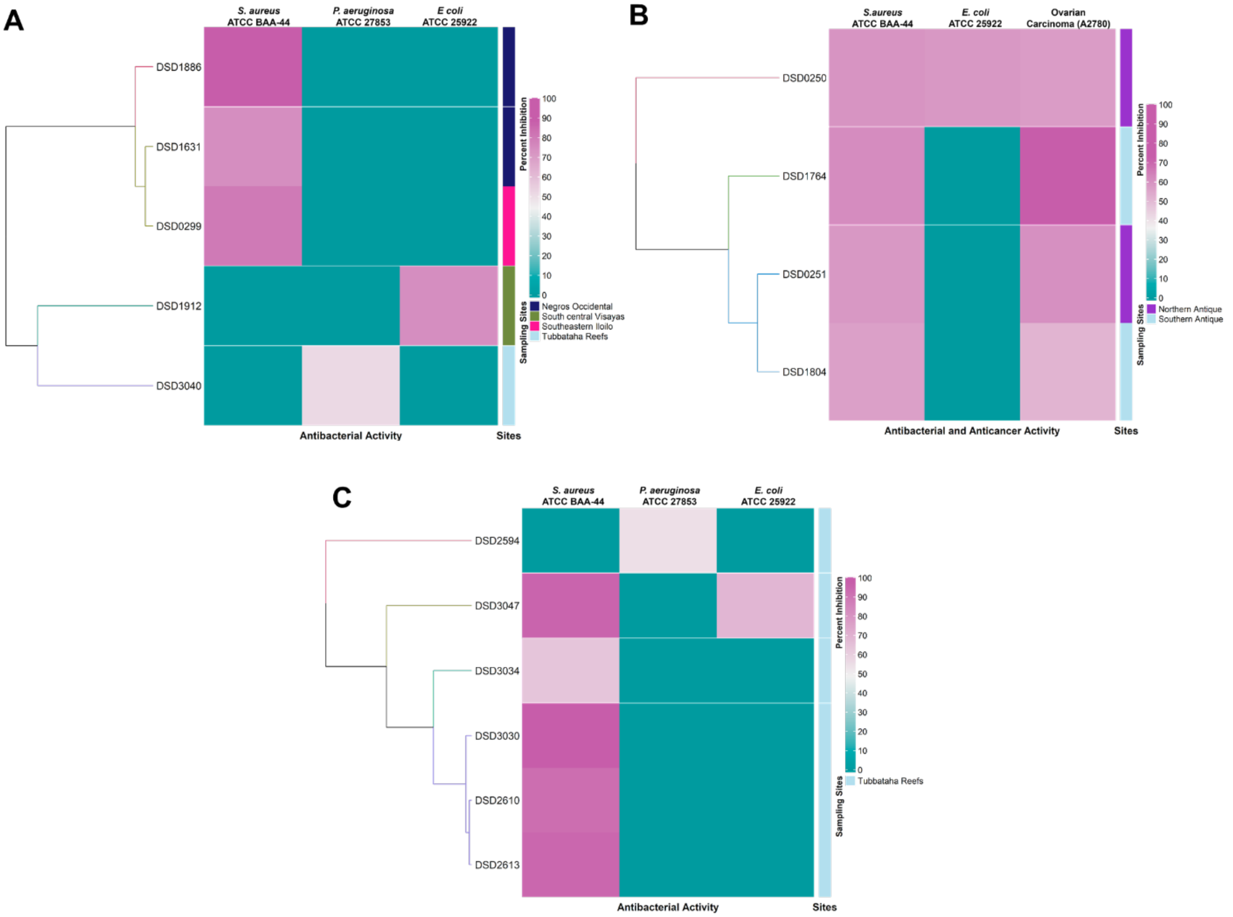

Supplementary Figure S4. Heat Map Based on the Antibacterial and Anticancer Activity of other Streptomyces species **(A)** ***S. diastaticus*****, (B)** ***S. kunmingensis,*** **and (C)** ***S. mutabilis,*** **against 4 test pathogens:** ***S. aureus*** **ATCC BAA-44,** ***E. coli*** **ATCC 25922,** ***P. aeruginosa*** **ATCC 27853*****, E. aerogenes*** **ATCC 13048.** Other *Streptomyces* species have shown strains belonging to the same species, found in the same site **(C)** or in different sites **(A)** and **(B)**, have variations in their antibacterial and anticancer activity. Strains belonging to the species *S. diastaticus* and *S. mutabilis* have not exhibited anticancer activity against ovarian carcinoma (A2780). Dendrogram on the left of the diagram indicate the relatedness of the strains based on the antibacterial and anticancer activities. Antibacterial and anticancer activity values range from 0-100% and correspond to a gradient of color steps ranging from blue-green (0%), white (50%), and purple (100%). Sampling sites are indicated by names and colors on the right side of the diagram. The heat map was created using the Rstudio statistical program ver1.2.5042 with heat map clustering methods, hierarchical clustering using a Euclidean distance metric to cluster the data.

A

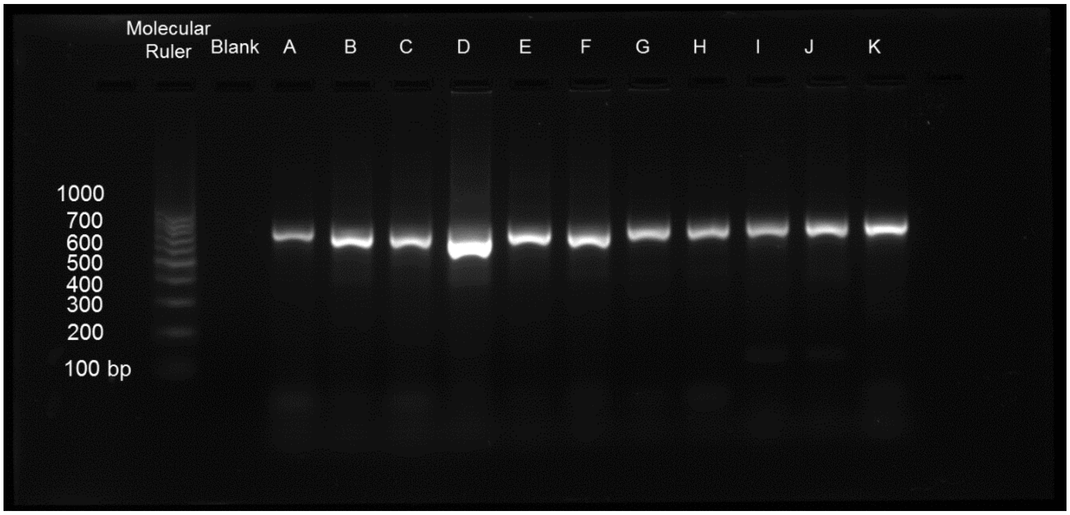

B

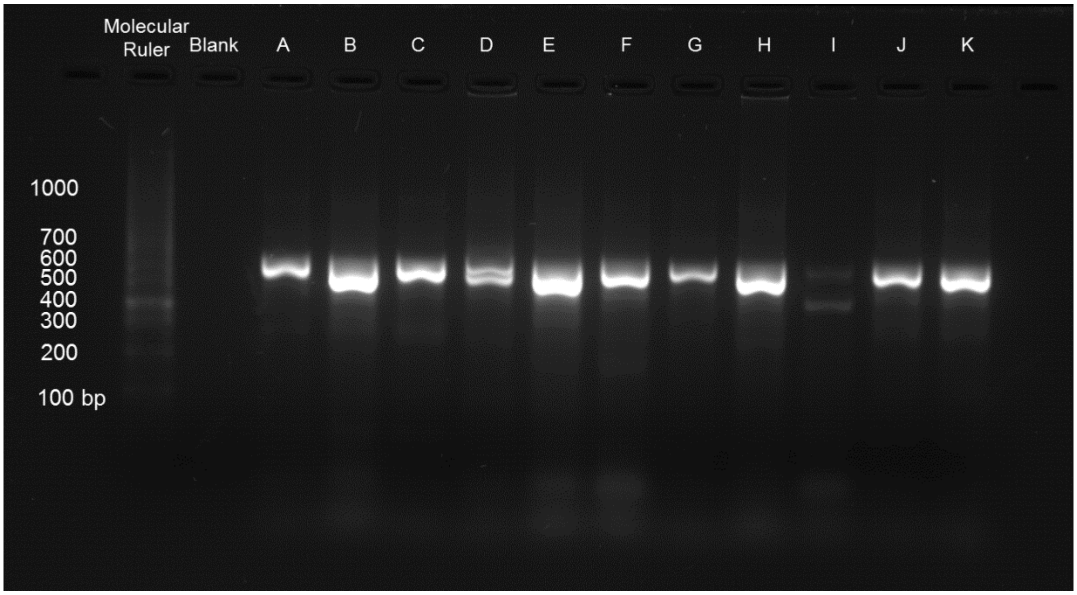

Supplementary Figure S5. Agarose Gel Electrophoresis of the NRPS Amplicons in *Streptomyces* sp. Strains **(A-B)**. Distinct band at 600-700 bp confirmed the presence of NRPS gene. Visualized using 2% agarose gel in 90V. **A)** Lanes A-K (A: *Streptomyces parvulus* strain DSD1632, B : *S. parvulus* strain DSD988, C: *S. parvulus* strain DSD1628, D: *S. parvulus* strain DSD154, E: *S. parvulus* strain DSD992, F: *S. parvulus* strain DSD1641, G: *S. mutabilis* strain DSD3034, H: *S. mutabilis* strain DSD3030, I: *S. sedi* strain DSD2987, J: *S. sedi* strain DSD3011, K: *S. diastaticus* strain DSD299). **B)** Lanes A-K (A: *Streptomyces ennisocaesilis* strain DSD318, B: *S. ennisocaesilis* strain DSD357, C: *S. olivaceus* strain DSD264, D: *S. pseudogriseolus* strain DSD2603, E: *S. cacaoi* strain DSD2595, F: *Streptomyces* sp. DSD3025, G: *S. kunmingensis* strain DSD250, H: *S. xiamenensis* strain DSD977, I: *S. harbinensis* strain DSD757, J: *S. rochei* strain DSD839, and K: *S. rochei* strain DSD2625).

A
B

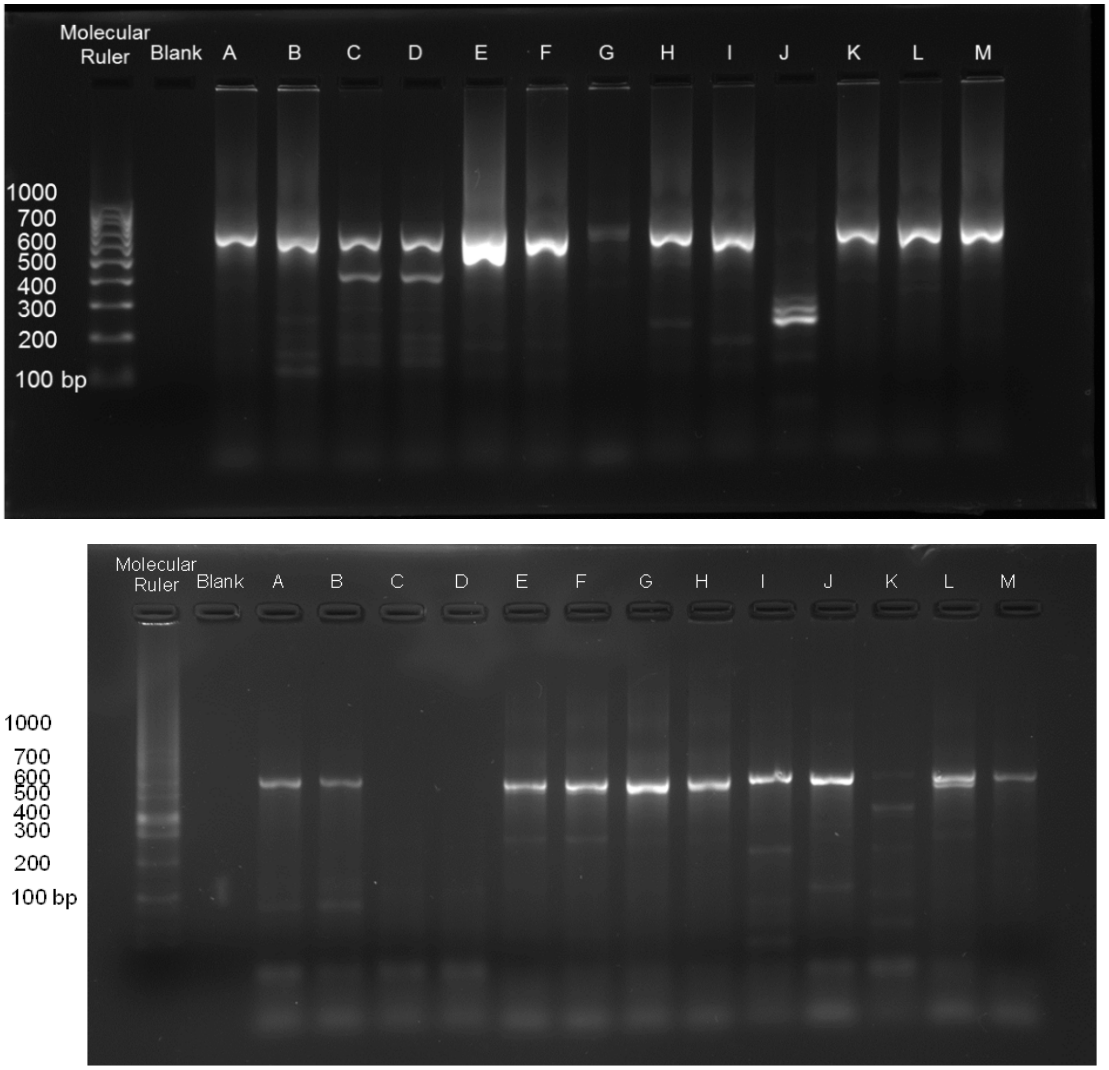

Supplementary Figure S6. Agarose Gel Electrophoresis of the PKS-I Amplicons of *Streptomyces* sp. Strains. Two sets of primers were used to amplify the β-ketoacyl synthetase domain of PKS-I. Fragments of 600-700 bp of PKS-I amplified by **A)** KSMA-F and KSMB-R primers. Lanes A-M (A: *Streptomyces parvulus* strain DSD993, B: *S. parvulus* strain DSD1628, C: *S. mutabilis* strain DSD3047, D: *S. mutabilis* strain DSD2613, E: *S. ennisocaesilis* strain DSD360, F: *S. ennisocaesilis* strain DSD357, G: *S. rochei* strain DSD1342, H: *S. rochei* strain DSD1328, I: *S. xiamenensis* strain DSD977, J: *S. diastaticus* strain DSD299, K: *Streptomyces* sp. DSD3025, L: *Streptomyces* sp. DSD742, M: *Streptomyces* sp. DSD1006). **B)** KS-F and KS-R primers. Lanes A-M (A: *Streptomyces parvulus* strain DSD1639, B: *S. parvulus* strain DSD985, C:- *S. mutabilis* strain DSD2610, D: *S. mutabilis* strain DSD3030, E: *S. rochei* strain DSD120, F: *S. ennisocaesilis* strain DSD318, G: *S. sedi* strain DSD3011, H: *S. pseudogriseolus* strain DSD2603, I: *S. geysiriensis* strain DSD476, J: *Streptomyces* sp. DSD3025, K: *Streptomyces* sp. DSD742, L: *S. albus* strain DSD1715, M: *S. kunmingensis* strain DSD1764). Gels were visualized using 2% agarose gel in 90V.

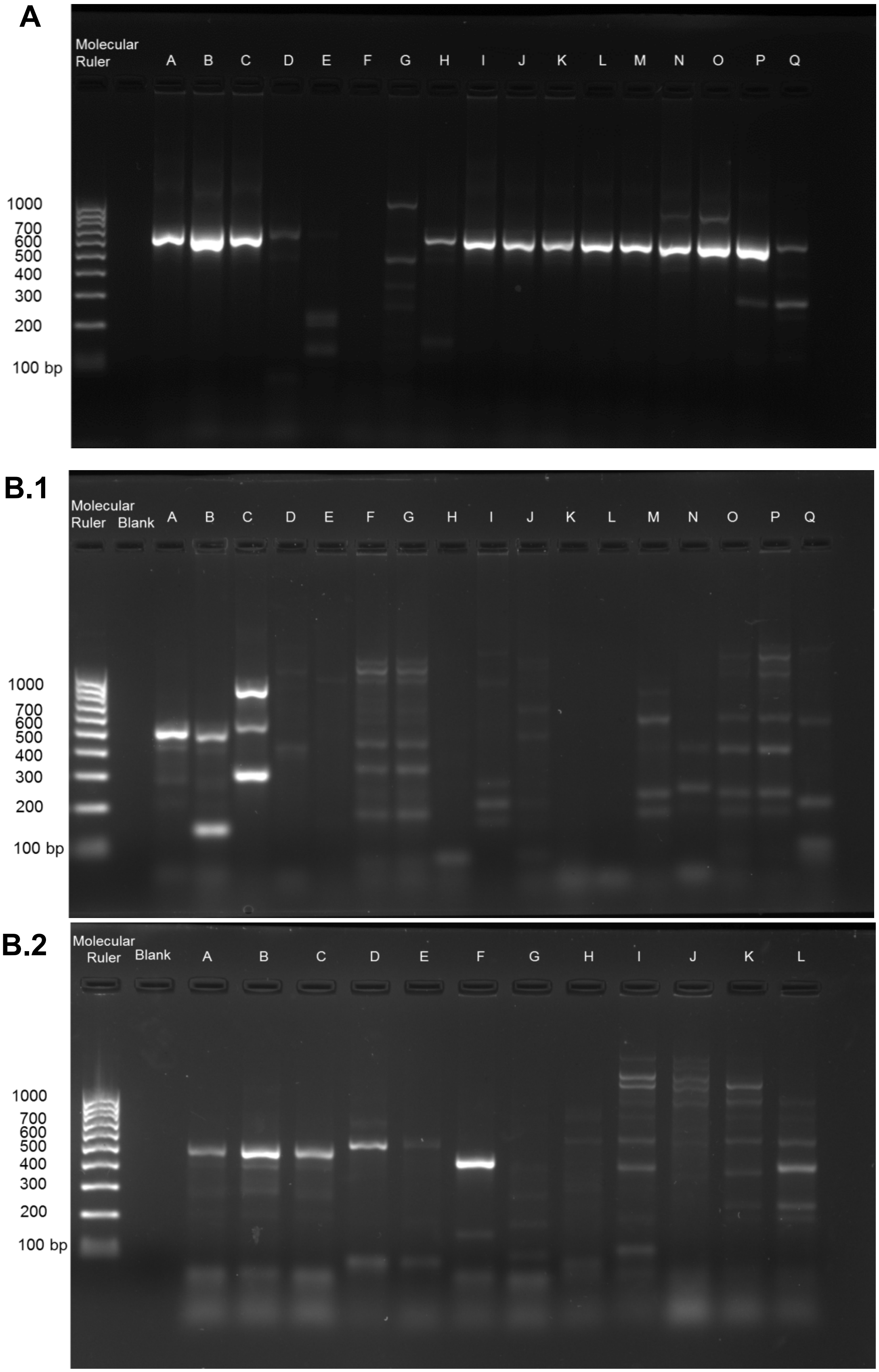
A
Molecular Ruler
A B C D E F G H I J K L M N O P Q
1000
700
600
500
400
300
200
100 bp
B.1
Molecular Ruler
Blank
A B C D E F G H I J K L M N O P Q
1000
700
600
500
400
300
200
100 bp
B.2
Molecular Ruler
Blank
A B C D E F G H I J K L
1000
700
600
500
400
300
200
100 bp

C

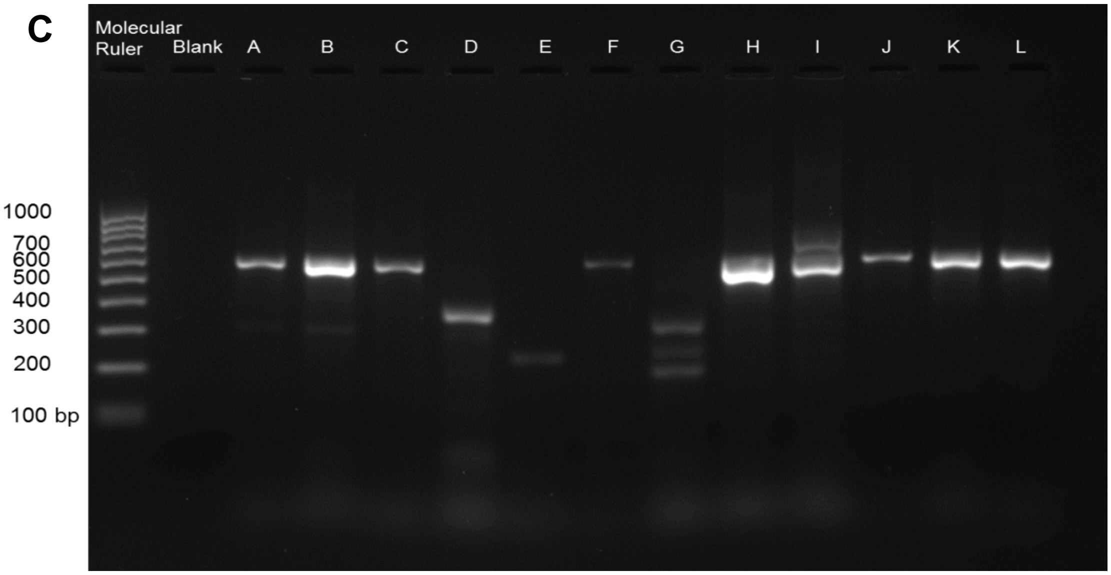

Supplementary Figure S7. Agarose Gel Electrophoresis of the PKS-II amplicons of *Streptomyces* sp. Strains. Three sets of primers were used to amplify type II PKS. **A**) KS1-F, KS1-R primers were used to target the ketosynthase α domain. Band size of 600 bp was observed. Lanes A-Q (A: *Streptomyces parvulus* strain DSD1629, B: *S. parvulus* strain DSD1643, C: *S. parvulus* strain DSD1642, D: *S. albus* strain DSD1715, E: *S. sedi* strain DSD3018, F: *S. sedi* strain DSD2987, G: *Streptomyces* sp. strain DSD3025, H: *S. xiamenensis* strain DSD977, I: *S. diastaticus* strain DSD1631, J: *S. mutabilis* strain DSD2613, K: *S. mutabilis* strain DSD2610, L: *S. geysiriensis* strain DSD176, M: *S. olivaceus* strain DSD618, N: *S. rochei* strain DSD1342, O: *S. rochei* strain DSD1555, P: *S. carpaticus* strain DSD331, Q: *S. carpaticus* strain DSD274). **B**) KS-α, KS-β targets ketosynthase (KS) α and KSβ domains which is indicated by a distinct band at 400-500 bp. **B.1**) Lanes A- Q (A: *Streptomyces parvulus* strain DSD988, B: *S. parvulus* strain DSD1072, C: *S. xiamenensis* strain DSD977, D: *S. sedi* strain DSD3018, E: *Streptomyces* sp. strain DSD1006, F: *S. diastaticus* strain DSD1886, G: S. *diastaticus* strain DSD1631, H: *S. diastaticus* strain DSD299, I: *Streptomyces* sp. strain DSD742, J: *S. albus* strain DSD1715, K: *S. mutabilis* strain DSD2610, L: *S. geysiriensis* strain DSD342, M: *S. enissocaesilis* strain DSD360, N: *S. enissocaesilis* strain DS1986, O: *S. rochei* strain DSD1328, P: *S. rochei* strain DSD1381, Q: *S. harbinensis* strain DSD757. **B.2)** A: *Streptomyces parvulus* strain DSD1628, B: *S. parvulus* strain DSD1641, C: *S. parvulus* strain DSD1690, D: *S. carpaticus* strain DSD331, E: *S. carpaticus* strain DSD274, F: *S. griseorubens* strain DSD767, G: *S. pseudogriseolus* strainDSD2603, H: *S. mutabilis* strain DSD3030, I: *S. enissocaesilis* strain DSD133, J: *S. enissocaesilis* strain DSD165, K: *S. rochei* strain DSD243, L: *S. rochei* strain DSD2625. **C**) Gene fragment at 554 bp indicates presence of 5' KSα domain of PKS-II. Lanes A-L (A: *Streptomyces parvulus* strain DSD1637, B: *S. parvulus* strain DSD1629, C: *S. xiamenensis* strain DSD977, D: *Streptomyces* sp. strain DSD3025, E: *S. albus* strain DSD1715, F: *Streptomyces* sp. strain DSD1006, G: *S. kunmingensis* strain DSD1764, H: *S. olivaceus* strain DSD618, I: *S. diastaticus* strain DSD1886, J: *S. mutabilis* strain DSD3034, K: *S. ennisocaesilis* strain DSD323, L: *S. rochei* strain DSD2625. Unspecific, multiple bands were considered negative. Visualized using 2% agarose gel in 90V.
